# Sky cooling for LED streetlights

**DOI:** 10.1038/s41377-024-01724-7

**Published:** 2025-02-26

**Authors:** Saichao Dang, Hasan H. Almahfoudh, Abdulrahman M. Alajlan, Hussam Qasem, Jiake Wang, Yingkun Zhu, Osman M. Bakr, Boon S. Ooi, Qiaoqiang Gan

**Affiliations:** 1 Sustainable Photonics Energy Research Laboratory, Material Science Engineering, PSE, King Abdullah University of Science and Technology (KAUST), Thuwal, 23955-6900 Saudi Arabia; 2 Future Energy Technology Institute, King Abdulaziz City for Science and Technology, Riyadh, 11442 Saudi Arabia; 3 Functional Nanomaterials Laboratory, Material Science and Engineering, PSE, KAUST, Thuwal, 23955-6900 Saudi Arabia; 4 Photonics Laboratory, Electrical and Computer Engineering, CEMSE, KAUST, Thuwal, 23955-6900 Saudi Arabia

**Keywords:** Optical techniques, Optical materials and structures

## Abstract

Thermal management is a critical challenge for semiconductor light-emitting diodes (LEDs), as inadequate heat dissipation reduces luminous efficiency and shortens the devices’ lifespan. Thus, there is an urgent need for more effective cooling strategies to enhance the energy efficiency of LEDs. LED streetlights, which operate primarily at night and experience high chip temperatures, could benefit greatly from improved thermal management. In this study, we introduce a sky-facing radiative cooling strategy for outdoor LED streetlights, an innovative yet less explored approach for thermal management of optoelectronics. Our method employs a nanoporous polyethylene (nanoPE) material that possesses both infrared transparency and visible reflectivity. This approach enables the direct release of heat generated by the LED through a sky-facing radiative cooling channel, while also reflecting a significant portion of the light back for illumination. By incorporating nanoPE as a cover for sky-facing LED lights, we achieved a remarkable temperature reduction of 7.8 °C in controlled laboratory settings and 4.4 °C in outdoor environments. These reductions were accompanied by an efficiency improvement of approximately 5% and 4%, respectively. This enhanced efficiency translates into substantial annual energy savings, estimated at 1.9 terawatt-hours when considering the use of LED streetlights in the United States. Furthermore, this electricity saving corresponds to a reduction of approximately 1.3 million metric tons of CO2 emissions, equivalent to 0.03% of the total annual CO2 emissions by the United States in 2018.

## Abstract (web summary)

Dang and Gan et al. reported a sky-cooling strategy for LED streetlights using a nanoporous polyethylene architecture that is highly transparent to infrared radiation and reflective in the visible spectrum.

## Graphical abstract

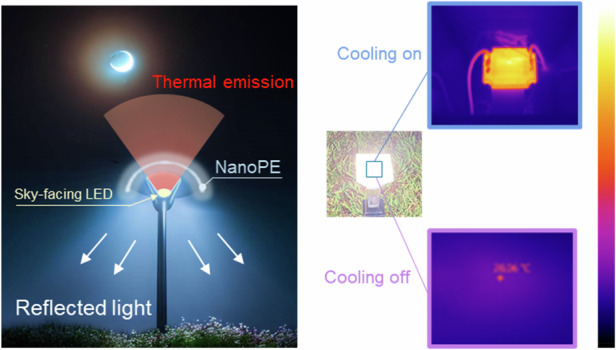

## Introduction

In the face of record-breaking temperatures and an increase in extreme weather events worldwide, it has become imperative for humanity to prioritize carbon emission reduction and foster the development of a sustainable Earth^1^. In this context, lighting plays a pivotal role, accounting for ~20% of the world’s annual electricity consumption^2^. The staggering consumption of around 4570 TWh in lighting alone during 2019 contributed to nearly 6% of global greenhouse gas emissions^3^. To combat these challenges and strive for carbon neutrality in the lighting sector, LED lighting has emerged as a critical solution, boasting superior energy efficiency and a longer operational lifespan compared to traditional incandescent lighting^4,5^. As part of this transition, inefficient incandescent light bulbs are officially being phased out in the US in August 2023, further driving the adoption of LED lighting as a more eco-friendly alternative^6^. However, despite their overall efficiency, LEDs, like other semiconductor devices such as solar cells^7^, encounter the issue of reduced efficiency and lifespan at high operating temperatures^8^. Notably, a significant amount of energy (70–80%) is still wasted as heat within LEDs^8^, leading to elevated chip temperatures and consequent degradation of efficiency and lifespan^8,9^. To bolster the performance-to-cost ratio of semiconductor lighting products, the development of more efficient thermal management strategies for high-power LEDs is imperative^10^. Numerous methods have been proposed to address this issue, including liquid cooling^11^, microchannel coolers^12,13^, and conventional heat sinks^14,15^. However, these techniques often necessitate additional manufacturing steps and materials, significantly increasing the cost of LEDs. Consequently, there is a pressing need for innovative, cost-effective, and streamlined thermal management solutions to optimize LED performance and foster sustainable lighting practices.

Recently, the potential of employing radiative cooling strategies for LED lights has been explored (e.g., refs. ^16–18^). It has been acknowledged that most commercial LED packages already incorporate coatings with high thermal emissivity on the backside^19^. Consequently, the radiative cooling potential for the backside of existing commercial LED products is limited. In contrast, the radiative cooling channel for the front side of the LED light exhibits a net enhancement compared to commercial products^20^. However, since most LED streetlights are designed to illuminate the street, the semiconductor chip can only transfer thermal radiation to the ground at the ambient temperature, which is close to 300 K. Thus, the full potential of sky cooling for LEDs remains untapped. Compared to extensively studied areas, such as building cooling with super-white roofing materials^21–25^ or personal thermal management using nano- and micro-fabric technologies^26–34^, the application of radiative cooling for high-temperature semiconductor devices remains relatively underexplored. While passive cooling strategies have been investigated for semiconductor photovoltaic (PV) devices, these systems often encounter practical challenges due to unavoidable solar heating during the day^7,35–39^. In contrast, LED streetlights, which primarily operate at night and are not exposed to solar irradiation, offer a distinctive opportunity for effective radiative cooling. Moreover, LED chips typically function at higher temperatures than solar cells, which suggests an even greater potential for radiative cooling effectiveness. Despite these promising aspects, the integration of radiative cooling into outdoor LED streetlight systems has not been thoroughly investigated, underscoring a novel research direction that bridges light science and applications with advanced thermal management strategies.

This article presents an architecture that enables sky cooling of the LED chip using engineered nanoporous polyethylene (nanoPE) films as a cover. By controlling the porosity and pore size of nanoscale and microscale pores within intrinsically mid-infrared (MIR) transparent polyethylene (PE) films, their optical scattering features can be engineered to reflect over 95% of visible light. Simultaneously, the films maintain over 80% transparency in the thermal radiation regime. This combined spectral selectivity enables direct sky cooling of LED streetlights. Both indoor and outdoor tests have been performed to demonstrate the effectiveness of using nanoPE as a cover for LEDs, resulting in a significant temperature reduction of 7.8 °C in laboratory conditions and 4.4 °C in outdoor settings, accompanied by an efficiency improvement of ~5–4%. This efficiency enhancement translates to substantial potential energy savings of 1.9 TWh in the United States, leading to a reduction of ~1.3 million metric tons of CO_2_ emissions, corresponding to 0.03% of the total annual CO_2_ emission by the US in 2018^40^. This new architecture for sky-facing LED chip design enables a thermal radiative pathway to the sky, thereby reducing material, labor, and maintenance costs associated with future global LED lighting products.

## Results

### General design

**Fig. 1:**
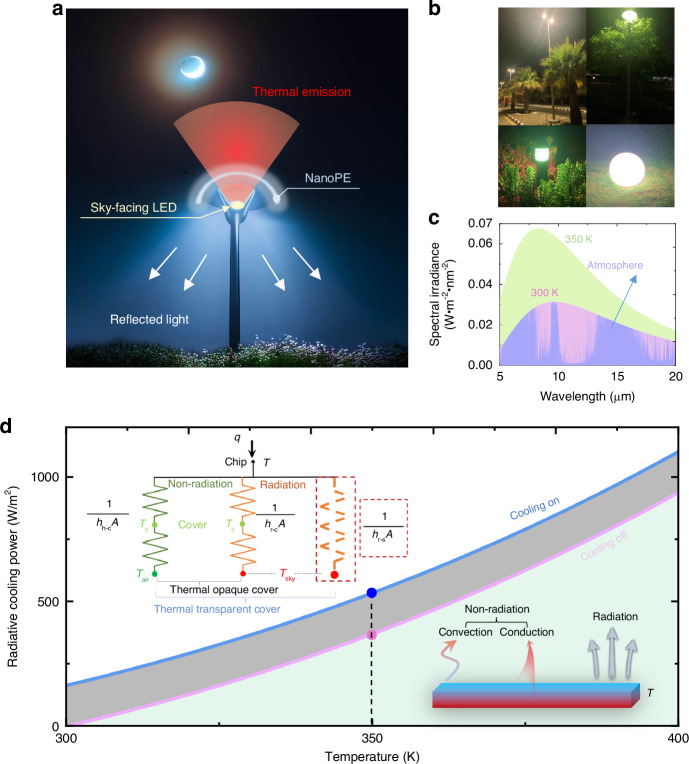
**Design of the sky-facing LED streetlight architecture**. **a** Schematic of the design for a sky-facing LED streetlight. **b** Representative outdoor LED light products used in the KAUST campus. **c** Spectral irradiance of blackbody at 300 and 350 K as well as the radiative heat flux between the blackbody and the atmosphere. The violet region represents the spectral irradiance of the atmosphere. The green region represents the radiative heat flux between a blackbody at 350 K and a blackbody at 300 K. The pink region represents the radiative heat flux between a blackbody at 300 K and the cold sky. **d** Cooling power when the LED has access to the sky through the nanoPE film (cooling on) compared to when no direct emission to the sky (cooling off). Inset: The corresponding heat transfer network with either a thermally opaque cover or a thermally transparent cover. For a thermally opaque cover, heat is transferred from the chip to the cover through both non-radiative and radiative processes, characterized by the coefficients *h*_h*−*c_ and *h*_r*−*c_, respectively. In contrast, a thermally transparent cover opens an additional radiative cooling channel from the chip to the sky, with the coefficient *h*_r*−*s_

Outdoor LED streetlights, responsible for 3–4% of global electricity consumption^41^, are extensively used for practical lighting due to their outstanding energy efficiency and cost-effectiveness, surpassing traditional incandescent lamps. However, conventional ground-facing LED streetlights are unable to fully leverage the cold sky for radiative cooling, as their thermally opaque covers impede the thermal radiation emitted by the LED chip. In response to this limitation, we propose a sky-facing LED streetlight design that capitalizes on the cold sky as a radiative cooling sink. In this configuration, the light emitted by the LED chip is directed upward towards the sky, instead of downward toward the ground. To further optimize radiative cooling, we introduce a cover composed of a nanoporous material that is transparent to infrared radiation while reflecting visible light (Fig. 1a). The primary objective of this design is to improve heat dissipation to the sky through a thermal radiation channel, leading to a reduction in the operating temperature of LED chips. Simultaneously, the new cover film also reflects most of the emitted light back to the ground for regular illumination. This dual functionality guarantees optimal performance and longevity of the LED lights, making them suitable for outdoor lighting applications (e.g., see Fig. 1b for several examples of outdoor LED streetlights in the KAUST campus). Considering that standard LED lights typically operate at temperatures ranging from 60 to 130 °C^8^, here we first analyze the radiative cooling potential of the sky-facing channel for LED chips.

According to Planck’s law, the overall thermal radiation of a blackbody is 459 W/m^2^ at 300 K. As shown by the combined green, violet and pink region in Fig. 1c, this value increases to 851 W/m^2^ at 350 K, 392 W/m^2^ higher than that from a blackbody at 300 K (i.e., the pink and violet region), revealing the potential of radiative cooling at higher surface temperatures (see calculation details in Note S1, Figs. s1, s2). Taking into account the non-radiative effects from the ambient environment on the LED, the sky-facing radiative cooling power during the nighttime (as shown in the “On” mode in Fig. 1d) can be calculated using

1 $${P}_{{\rm{cool}}}(T)={P}_{{\rm{rad}}}(T)-{P}_{{\rm{atm}}}({T}_{{\rm{atm}}})-{P}_{{\rm{cond}}+{\rm{conv}}}(T)$$

Here *T* and *T*_atm_ are the temperatures of the LED and atmosphere, respectively. *P*_rad_ is the thermal radiation of the LED, which is the fourth power of its temperature multiplied by the Stefan–Boltzmann constant. *P*_atm_ is the absorbed thermal radiation by the object from the atmosphere (violet region in Fig. 1c). *P*_cond+conv_ is the heat load of the emitter due to the conductive and convective heat exchange with ambient, which can be characterized by a combined heat transfer coefficient (*h*_h–c_), as shown in the heat transfer network depicted in the inset of Fig. 1d (see details in Note S2, Figs. s3, s4). This network illustrates the overarching thermal management functionality of a thermally transparent cover. Specifically, in comparison to a thermally opaque cover, a thermally transparent film adds an extra radiative thermal resistance between the chip and the sky, represented by 1/(*h*_r–s_*A*) in the red dashed frame. This leads to a reduction in the overall thermal resistance of the entire system, resulting in improved cooling performance. For example, when an LED chip operates at 350 K at its surface under an ambient temperature of 300 K (see modeling setting details in Note S2), the sky-facing architecture demonstrates a remarkable net cooling power of 536 W/m^2^ (shown as the blue dot in Fig. 1d). This value surpasses the cooling power obtained without the sky access (pink dot in Fig. 1d) by 164 W/m^2^. This highlights the effectiveness of our design for outdoor LED lights as it allows the release of thermal radiation to the cold sky without compromising illuminating performance. In Note S3 (Fig. s5, Tables s1 and s2), we evaluated a commercially available LED streetlight, measuring the temperatures of its front and back surfaces. We then compared the overall cooling powers between the conventional ground-facing configuration and our proposed sky-facing setup. This analysis demonstrated an enhanced cooling power potential of up to 510 W/m^2^ (refer to Table s1), further validating the efficacy of the proposed sky-facing cooling channel. However, a key challenge lies in directing the emitted light from the sky-facing LED chips for ground-facing illumination purposes.

To achieve this simultaneous light redirection and enhanced radiative cooling, we propose the utilization of nanoPE films, which allow for the combination of high visible reflection and high thermal radiation transmission. Polyethylene (PE), a polymer extensively used in various practical applications, has gained considerable attention in the field of radiative cooling due to its remarkable optical properties in both the visible and MIR spectral bands^42–44^. The high transmittance in the MIR region is attributed to the low absorption characteristics of the polyethylene backbone, which is composed primarily of aliphatic C–C and C–H bonds^45–47^. When engineered with a nanoporous structure, nanoPE further enhances its optical performance. The introduction of nanopores creates a refractive index mismatch between air and PE, which significantly amplifies light scattering. This scattering effect leads to high reflection in the visible spectrum, effectively redirecting emitted light upwards while simultaneously allowing thermal radiation to pass through. Importantly, the pores in nanoPE are much smaller than the wavelength of mid-infrared light (~10 μm), ensuring that the film retains high transmittance in this crucial spectral range. This characteristic allows the LED chip to radiate heat efficiently to the cold sky, thereby improving the thermal management of the streetlight (refer to the next section for a detailed discussion on the optical and thermal properties of nanoPE). Previously reported pioneering studies (e.g., refs. ^26,27,30,31,34,48^) have utilized various nanoPE films, including commercially available products^26^, as new materials for white fabrics, thereby enhancing thermal management for the human body. The primary focus of those studies has been to leverage the high transmission of thermal radiation to facilitate radiative cooling for human bodies where the incident solar light is scattered randomly to minimize the solar heating effect. In other words, the visible light was unwanted and was intentionally disregarded (e.g. refs. ^26,27,30,31,34,48^). As a result, radiative cooling is usually exclusive to light or solar energy usage (e.g. refs. ^26,27,30,31,34,48^). However, in the current study, we aim to investigate this material and explore its application in achieving radiative cooling in sky-facing LED lighting systems. In this particular application, the visible light emitted by the LED products is essential for directional illumination purposes (e.g., ground-facing streetlights), which cannot be simply reflected in the sky nor scattered randomly to the surrounding environment. Therefore, by exploring the potential of nanoPE films in sky-facing radiative cooling for LED streetlights, we seek to bridge the gap between enhanced cooling efficiency and efficient illumination and realize concurrent radiative cooling and light management for future more sustainable LED lights.

### NanoPE fabrication and characterization

By introducing nano-sized pores into the PE material, we can achieve high reflectance in the visible range while maintaining excellent transmittance in the MIR region (e.g., refs. ^27,30,31,34,48^), simultaneously. In this study, we used two main methods to demonstrate scalable manufacturing of nanoPE films.

**Fig. 2:**
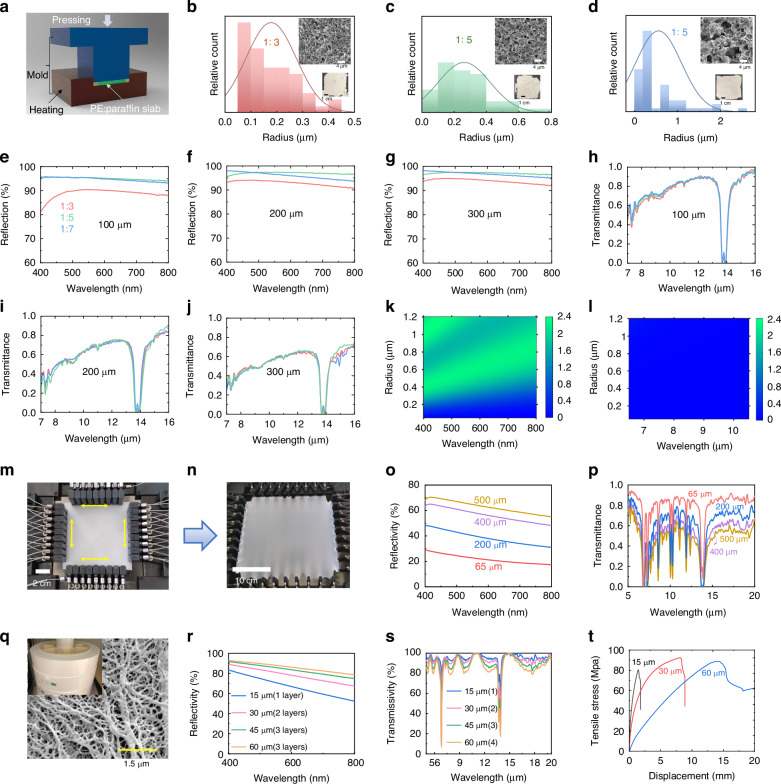
**NanoPE fabrication and characterization**. **a** Schematic of hot-press to fabricate nanoPE. **b**–**d** Pore-size distribution of nanoPE films with mixing ratios of 1:3, 1:5, and 1:7 (inset shows the corresponding SEM images and photos of samples). **e**–**g** Visible light reflection of nanoPE films with different thicknesses and mixing ratios. **h**–**j** MIR transmittance of nanoPE films with different thicknesses and mixing ratios. **k** Scattering efficiency of nano-sized pores in the visible band by Mie scattering theory. **l** Scattering efficiency of nano-sized pores in the mid-infrared band by Rayleigh scattering theory. **m** Biaxial orientation stretching machine with PE film. **n** The BOPE film in dry process. **o** The visible reflectance of BOPE film with different thicknesses. **p** The mid-infrared transmittance of BOPE film with different thicknesses. **q** SEM of BOPE in the wet process as well as the scalable nanoPE. **r** The visible reflectance of scalable nanoPE with different thicknesses. **s** The mid-infrared transmittance of scalable nanoPE with different thicknesses. **t** Tensile stress of nanoPE films with different thicknesses

The first method involves using a hot-press technique to compress a mixed PE-paraffin-oil slab, as visualized in Fig. 2a. Through the removal of paraffin oil using methylene chloride (see the “Materials and methods” section for more technical details), this process results in the creation of nanopores within the PE film. Specifically, we adjusted the value of PE:oil ratio (i.e., the volume of paraffin oil in milliliters to the total weight of PE in grams) to fine-tune the pore size distribution. As illustrated in Fig. 2b–d, changing the PE:oil ratio from 1:3 to 1:7 results in an increase in pore radius from 177 to 550 nm. Increasing the amount of paraffin oil results in larger pores, as the oil is extracted from the PE film, leaving behind corresponding pores. These nanopores are responsible for substantial scattering of visible light, as exemplified by the white sample shown in the inset of Fig. 2b–d. Figure 2e–g demonstrates that nanoPE films with different thicknesses achieve visible light reflection higher than 0.8. The nanoPE with a 1:3 ratio, having smaller pores and weaker scattering effects, exhibits lower visible reflection compared to nanoPE film with 1:5 and 1:7 ratios of the same thickness. The nanoPE film with a 1:5 ratio shows slightly higher visible reflection due to its dense pores with a radius of around 255 nm (see the SEM in Fig. 2c). As the thickness of the nanoPE film increases from 100 to 300 μm, visible reflection slightly increases, as shown in Fig. 2e–g. As a result, the visible reflection of nanoPE is controlled by the nanoscale pores, which, due to their size being comparable to visible wavelengths, induce a strong scattering effect.

In contrast, the nanoscale pores exhibit a weaker scattering effect in the MIR band. The MIR transmittance of nanoPE is primarily influenced by its thickness. For instance, in the case of nanoPE with a 1:5 ratio, increasing the film thickness from 100 to 300 μm results in a reduction in average MIR transmittance from 0.81 to 0.55 (see the green curves in Fig. 2h, i). In conclusion, the introduction of nano-sized pores in this design primarily aims to improve backscattering of visible light. These pores are much smaller than the wavelength of thermal radiation, which means their presence does not significantly affect thermal transmittance.

To validate this principle, we further use classic scattering theory to investigate the scattering effects of pores of various sizes in both the visible and MIR bands. The nano-sized pores are designed to be comparable in size to the wavelength of visible light, which is critical for achieving strong reflection in that range. This scattering effect in the visible spectrum can be modeled using Mie scattering theory^49^, as depicted in Fig. 2k. In the mid-infrared band (7–15 μm), the pore size is significantly smaller than the wavelength, causing Rayleigh scattering to become the dominant scattering mechanism. This can be calculated using the Rayleigh scattering equation^49^:

2 $${Q}_{{\rm{sca}}}=\frac{8}{3}{\left(\frac{2\pi r}{\lambda }\right)}^{4}{\left(\frac{{m}^{2}-{m}_{0}^{2}}{{m}^{2}-2{m}_{0}^{2}}\right)}^{2}$$

where *r* is the pore radius, *λ* is the wavelength of light in the medium, *m* is the refractive index of the pores, and *m*_0_ is the refractive index of the surrounding medium (see more details in Note S4). Although Rayleigh scattering predominates in the mid-infrared band, the overall scattering intensity (Fig. 2l) is generally lower than that observed with Mie scattering when the pore size is comparable to the wavelength (Fig. 2k). Consequently, the scattering efficiency of nano-sized pores in the visible band is much higher than in the mid-infrared band. This results in nanoPE exhibiting high reflectivity in the visible band, while maintaining high transmissivity in the MIR band.

To enhance thermal conductivity and better harness radiative cooling capabilities, we implemented a second method known as the biaxial orientation stretching process to manufacture slim nanoPE films, commonly referred to as biaxially oriented PE (BOPE). This approach is particularly advantageous for its potential for large-scale mass production. One of its key merits lies in its capacity to align and arrange polymer chains parallel to the film plane, resulting in improved mechanical properties and thermal conductivity (i.e., from 0.2 to 0.9 W/(m K), see Note S5 for details, Figs. s6, s7, Table s3), encompassing impact strength, bending strength, tensile strength, and elastic modulus^50^. This technique has been effectively employed to enhance the mechanical attributes of various polymers, including polypropylene (PP), PE, and polyethylene terephthalate (PET)^50–53^. As depicted in Fig. 2m and n, we subjected a 10 cm × 10 cm × 0.1 cm PE film to stretching, transforming it into a larger and thinner 27 cm × 27 cm × 65 μm sheet (as detailed in Note S6, Figs. s8, s9). Subsequently, the film became thinner and exhibited nanopores, as shown in Fig. s9. However, the presence of nanopores was less pronounced, leading to relatively modest reflection in the visible spectrum (Fig. 2o) compared to the nanoPE produced using the first method (Fig. 2e–g). The BOPE film, generated through a dry process and measuring 65 μm in thickness, exhibited an ~25% reflectance in the visible domain. As the film thickness was adjusted from 65 to 500 μm, the reflection increased from 0.25 to 0.64, while MIR transmittance decreased from 0.90 to 0.53, as demonstrated in Fig. 2p. It is evident that reduced MIR transmission is suboptimal for radiative cooling applications.

To achieve a balance between high MIR transmission and robust visible light reflection, a hybrid approach can be adopted by combining the BOPE film created through the dry process with the thermal extraction process from the first method. However, it is important to note that this approach typically requires costly industrial equipment, which is often beyond the reach of most research groups. In our case, we leveraged an industrial partner to fabricate these nanoPE films using the advanced BOPE process (Fig. 2q). For a detailed account of the customized manufacturing process, please refer to Note S7 and Fig. s10. The pore size distribution analysis of the nanoPE film reveals that it predominantly features pores ranging between 30 and 120 nm in size, which is effective in scattering visible light (see Note S8 for characterization details, Fig. s11). The resultant nanoPE film boasts a thickness of ~15 µm and showcases remarkable reflectance values exceeding 80% in the visible spectrum (Fig. 2r), coupled with impressive MIR transmittance values surpassing 95% (Fig. 2s). Nonetheless, it is worth noting that this particular type of nanoPE film may not be sufficiently thick to entirely block visible light. To address this limitation, we conducted optical property measurements for folded films at various thicknesses, as depicted in Fig. 2r for the visible spectrum and Fig. 2s for the MIR range. A detailed analysis of the impact of thickness on the optical properties can be found in Note S9 and Fig. s12. Notably, this type of nanoPE film exhibits exceptional strength, surpassing that of conventional LED light cover materials like PMMA (with a tensile strength typically ranging between 30 and 50 MPa^54,55^). In Fig. 2t, we present the tensile stress characterization of our nanoPE film, with more comprehensive details available in Note S10 and Fig. s13. The results demonstrate that these films are capable of withstanding stresses up to 81–92 MPa before experiencing rupture, as shown in Fig. 2t. Furthermore, increasing the thickness of the nanoPE film enhances its ability to endure even greater forces, rendering it highly practical for various lighting applications (see more discussion in Note S10, Figs. s14, s15). In the subsequent sections, we will employ this engineered nanoPE film as a cover to demonstrate the efficacy of sky-facing radiative cooling for outdoor LED streetlights.

### Indoor radiative cooling performance

**Fig. 3:**
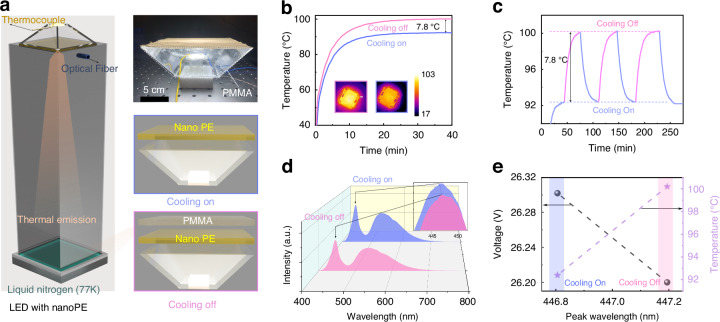
**Indoor experimental results of radiative cooling for LED light**. **a** Schematic of the indoor experiment as well as the in-situ image of the LED light. For cooling on, the LED chip is covered by a layer of nanoPE, while the chip is covered by nanoPE and PMMA for cooling off. Transparent polyethylene cover is used in both setups to reduce parasitic heat loss. **b** LED temperature and thermal images of the LED in “cooling on” (blue) and “cooling off” (pink) modes in the indoor setup. **c** The measured temperature of the LED light in “cooling on” and “cooling off” modes. A temperature reduction of ~7.8 °C is obtained. **d** The spectral intensity of the emitted light from the LED chip. The inset shows the blue-shift in the case of “cooling on”. **e** Voltage and temperature vs. peak wavelength at cooling on and cooling off

To evaluate the cooling performance of our design, we conducted an indoor test in a controlled laboratory environment. The test was conducted under an ambient temperature of ~20 °C and a relative humidity (RH) of ~60%. As shown by the left panel of Fig. 3a, the test setup consisted of a commercially available LED chip with a correlated color temperature (CCT) of 6700 K, enclosed within a 4-walled triangular chamber made of PMMA. The ceiling of the chamber was made of a nanoPE film, positioned about 6 cm from the LED chip (refer to Note S11 and Fig. s16 for a detailed discussion of how this gap affects temperature and illuminated area). In this indoor setting, a highly thermal emissive surface was employed as the remote heat sink, which was immersed in liquid nitrogen (LN) at a temperature of 77 K (e.g., ref. ^56^ see more details about the indoor settings in Note S12 and Figs. s17, s18). By facing the LED chip to this cold surface, efficient dissipation of heat through radiative cooling was facilitated. The main objective of the experiment was to compare the temperatures achieved in two scenarios (as illustrated by right-side panels of Fig. 3a): (1) When the LED was intercepted by a layer of nanoPE film (cooling on), the system would allow thermal transmission. (2) When the LED was intercepted by both nanoPE and PMMA films (cooling off), the thermal radiation would be blocked while not affecting visible light illumination. By analyzing the temperature differences under these different conditions, we were able to assess the effectiveness of our design in enhancing heat dissipation.

As illustrated in Fig. 3b, the LED chip’s temperature exhibits a gradual rise until it reaches a steady state after ~40 min of operation. With an applied current of 0.2 A, the LED temperature stabilizes at 100.1 °C in the “cooling off” mode, while in the “cooling on” mode, it attains a lower temperature of 92.3 °C, resulting in a notable temperature reduction of 7.8 °C (refer to Note S13 and Fig. s19 for the corresponding junction temperature). This pronounced cooling effect is readily observed using a thermal imager, as depicted in the inset of Fig. 3b. To ascertain the consistent performance of radiative cooling, we conducted three cycles of switching the cooling mode “On” and “Off,” as presented in Fig. 3c. Notably, the temperature reduction achieved through radiative cooling remains stable throughout the cycles, underscoring the potential of radiative cooling for LED lighting applications. Furthermore, this temperature reduction has a significant impact on the light emission characteristics of the LED, which were analyzed using a spectrometer equipped with an optical fiber facing the LED chip (highlighted in Fig. 3a) (see Note S14 and Fig. s20 for the characterization details). Figure 3d illustrates that under identical driving conditions, the intensity of light emitted from the LED chip increases when the “cooling on” mode is enabled. Additionally, the blue peak of the light spectrum shifts from 447.2 to 446.8 nm (see Note S15 and Fig. s21 for detailed information). This spectral shift is a direct consequence of the temperature reduction achieved by the semiconductor chip. Simultaneously, the input voltage experienced a marginal increase from 26.20 to 26.30 V (Fig. 3e), which is caused by the reduction of LED temperature (i.e., from 100.1 to 92.3 °C)^57^. Consequently, the efficiency of the LED light is enhanced by ~4.9% when the LN temperature (77 K) is employed as the cold source, considering the input power and output light intensity (see detailed calculations in Note S16). However, it is crucial to acknowledge that outdoor implementation is subject to numerous uncontrollable environmental conditions, necessitating further validation of the practical cooling effect, as will be discussed in subsequent sections.

### Outdoor experiment

**Fig. 4:**
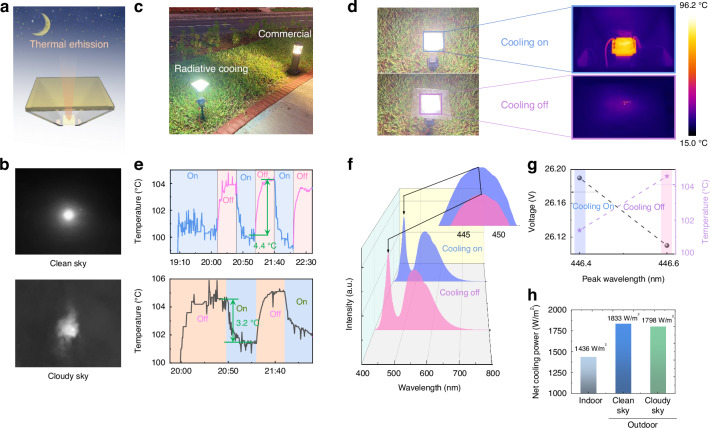
**Outdoor experimental results of sky-facing LED light**. **a** Schematic of the outdoor experiment. **b** Images of the clear sky and cloudy sky at night. **c** Outdoor experiment demonstration. Comparison of the LED light with radiative cooling and the commercial one in a park. **d** Visible image of the setup in different cooling modes and their corresponding IR image (right panel). **e** The measured temperature of the LED light in “cooling on” and “cooling off” modes under clean sky and cloudy sky. A temperature reduction of ~4.4 and ~3.2 °C are obtained under clear sky and cloudy sky, respectively. **f** The spectral intensity of the emitted light from the LED chip under a clear sky. The inset shows the blue-shift in the case of “cooling on”. **g** Voltage and temperature vs. peak wavelength at cooling on and cooling off under a clear sky. **h** The net cooling powers of indoor and outdoor experiments (i.e. under the clear sky and cloudy sky) of the cases “cooling on”

The outdoor experimental setup is illustrated in Fig. 4a and was conducted on May 8–9, 2023, within the campus of King Abdullah University of Science and Technology (at Thuwal, Saudi Arabia) (see more details about the outdoor settings in Note S12). Throughout the experiment over two days with different sky conditions (i.e., clean and cloudy as shown in Fig. 4b), the ambient temperature hovered around 27.5 °C, with relative humidity (RH) of ~60% (see detailed weather data in Note S17, Fig. s22, s23). As depicted in Fig. 4c, the custom-designed LED light demonstrated comparable illuminance to a commercial outdoor LED streetlight in a park (refer to Note S18 and Fig. s24 for data on the radiative cooling effect on a commercial LED lamp). To verify the effectiveness of radiative cooling, we conducted periodic alternations between the “cooling on” and “cooling off” modes. For this purpose, a PMMA slab was introduced above the nanoPE layer to obstruct the thermal radiation emitted by the LED chip. Figure 4d exhibits a close-up thermal image wherein the heated LED chip is clearly observable through the nanoPE film, highlighting the sky-facing radiative cooling channel integrated into our novel architecture. Conversely, in the “cooling off” mode, the thermal radiation is not evident through the PMMA film. Repeated switching between the two modes was carried out at 30-min intervals to demonstrate the consistent cooling effect, as illustrated in Fig. 4e. The temperature difference between the two modes was ~4.4 °C (i.e., from 104.2 to 99.8 °C, see the upper panel) under the clear sky and 3.2 °C under the cloudy sky (i.e., from 104.6 to 101.4 °C, see the lower panel). As shown by the spectral measurement results in Fig. 4f, the blue peak of the LED chip shifted from 447.6 to 447.0 nm under the clean sky, confirming the reduced operational temperature of the LED chip. By using the light intensity of the emitted light (e.g., the intensity increase of 4.4% under identical driving conditions under the clean sky, Note S14) and the junction voltage (increased from 26.11 to 26.20 V under the clean sky, Fig. 4g), the efficiency of the LED light is estimated to be increased by 4.4% (see calculation details in Note S14 and detailed characterization under the cloudy sky in Note S19, Figs. s25, s26).

Although the absolute value of the temperature reduction is slightly lower than that observed in the indoor experiment, the actual cooling power introduced by the sky-facing cooling channel is heavily dependent on the actual operational temperature of the LED chip. It is essential to consider that the ambient temperature in outdoor conditions is approximately 27.5 °C, significantly higher than the indoor temperature of 20 °C. This temperature difference inevitably impacts the operational temperature of the LED chip during outdoor testing. Additionally, despite the low temperature of outer space (~3 K) under ideal conditions, various environmental factors such as humidity, cloud distribution, air pollution, and wind will affect the practical cooling performance, leading to a much higher effective cold source temperature (e.g., 40 K lower than the ambient^58–60^). The temperature reduction cannot be simply used as a fair figure-of-merit for direct comparison. Instead, a more appropriate parameter to assess the cooling performance is the net cooling power, which measures 1436 W/m^2^ for the indoor experiment (based on the data shown in Fig. 3), 1833 and 1798 W/m^2^ for the outdoor experiment under a clear and cloudy sky, respectively (Fig. 4h, see calculation details in Note S20). These experimental findings underscore the true potential of the sky-facing cooling channel in terms of energy efficiency and carbon emission reduction. Leveraging such a cooling mechanism can lead to significant energy and environmental benefits.

**Fig. 5:**
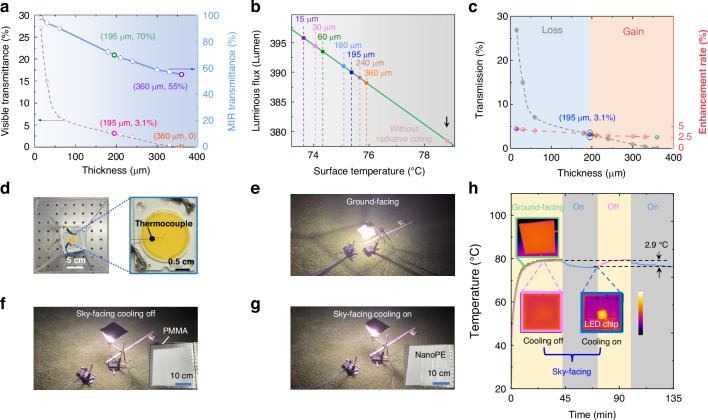
**NanoPE films with zero upward light leakage**. **a** Averaged visible transmittance and MIR transmittance as a function of the nanoPE film thickness. **b** Cooling effect of nanoPE films with varying thicknesses and their associated luminous flux levels. **c** The thickness-dependent transmission through the nanoPE film (the gray dashed line) and the corresponding enhancement rate of the luminous flux compared to the no radiative cooling effect (the red dashed line). **d** Demonstration of setup and illumination under conditions of ground-facing (**e**), sky-facing cooling off (**f**), and sky-facing cooling on (**g**). **h** The outdoor cooling performance tests and corresponding thermal images

In response to increasing global regulations on light pollution, which prohibit upward light leakage in many areas, it is essential to control light emission both vertically and horizontally, as reflected in the legislation of leading countries^61–63^. To align with these environmental mandates, we then investigated the optical and thermal transmission properties of nanoPE films at different thicknesses. Our findings, detailed in Fig. 5a, show that a thickness of 195 µm reduces optical transmission to 3.1% while maintaining high thermal transmission at 70%. At 360 µm, optical transmission is nearly blocked, with thermal transmission decreasing to 55% (see details in Note S21 and Note S9, Fig. s27). To assess the nanoPE film’s effectiveness further, we analyzed the luminous flux in relation to the surface temperature of an LED chip under a 0.1 A current, using a commercial temperature-controllable integrating sphere system (Fig. s19). This analysis helped establish a quantitative relationship between luminosity and chip surface temperature reduction (Fig. 5b, see details in Note S22, Fig. s28). Outdoor tests then measured the chip’s surface temperature with nanoPE films of various thicknesses, allowing us to evaluate their cooling effects compared to the control condition with no radiative cooling effects (a surface temperature of 78.8 °C, marked by the black arrow in Fig. 5b). These tests indicated a net benefit for film thicknesses exceeding 195 µm. With a 360-µm-thick nanoPE film to block the upward light leakage completely, the system exhibits a luminous flux of 388.2 lumens, representing a gain of 2.5% (i.e., the green dot in Fig. 5c) compared to no radiative cooling (i.e., 378.6 lumens marked by the black arrow in Fig. 5b). Our subsequent discussion will explore the benefits of this zero-optical-transmission film in outdoor settings.

To mitigate upward light transmission and prevent light pollution, we utilized an LED chip with a CCT of 3000 K, combined with a 360-μm-thick nanoPE film, as demonstrated in Fig. 5d. This setup was tested outdoors on the KAUST campus on the evening of March 8, 2024, under ambient conditions of ~22 °C and 60% relative humidity (see more details about the outdoor settings in Note S12). The experiment involved the LED chip under three distinct cover setups (Fig. 5e–g), all operating at a 0.1 A current. The first configuration, shown in Fig. 5e, employed a ground-facing orientation with a 0.5 mm-thick transparent PMMA cover, mimicking traditional LED lighting setups. In this scenario, the LED chip’s temperature steadily rose, stabilizing at 79.2 °C after around 45 min (green line in Fig. 5h). Subsequently, the setup was adjusted to face the sky, as depicted in Fig. 5f, where a 360-µm-thick nanoPE film was placed beneath the PMMA cover to direct all emitted light downward, effectively entering a cooling-off mode. This adjustment resulted in the LED chip maintaining a temperature of 78.8 °C (pink line in Fig. 5h), roughly mirroring the temperature of the ground-facing setup with the radiative cooling channel blocked by the PMMA cover. Most notably, removing the PMMA cover to expose the LED to the sky directly (Fig. 5g) activated the radiative cooling channel, leading to a significant temperature drop of 2.9 °C, from 78.8 to 75.9 °C (blue line in Fig. 5h). Reapplying and then removing the PMMA cover demonstrated a consistent ability to manipulate the operational temperature, confirming the radiative cooling effect’s efficacy. The insets in Fig. 5h provide thermal imagery of the setups, clearly showing the LED chip through the 360-µm-thick nanoPE film and affirming the substantial cooling effect facilitated by the nanoPE cover. Interestingly, the introduction of the nanoPE cover will alter the light intensity distribution. As shown in Fig. 5e, we captured the light intensity distribution from a ground-facing streetlight. In contrast, due to its highly scattering properties, the nanoPE film resulted in a more uniform light distribution compared to traditional ground-facing streetlights (see Fig. 5f and g). For a more detailed quantitative analysis, please refer to Note S23, Fig. s29.

### Practical conditions in extreme environments

**Fig. 6:**
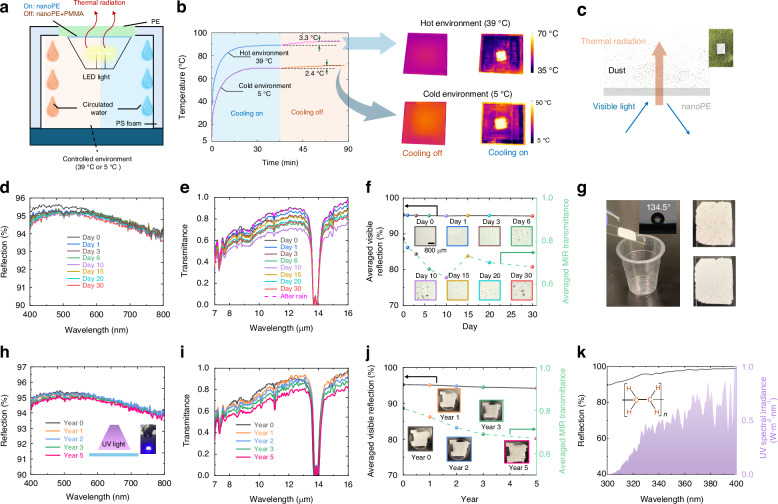
**Cooling performance in extreme temperatures and the impact of practical conditions on the nanoPE film**. **a** Schematic of the experimental setup in extreme temperatures. **b** LED chip temperature in hot and cold environments with cooling on and off, as well as the corresponding thermal images. **c** The 100 µm-thick nanoPE film was exposed outdoors at the KAUST campus. **d** Visible reflection of the nanoPE film on the bottom side under outdoor exposure. **e** Mid-infrared transmittance of the nanoPE film under outdoor exposure. **f** Average visible reflection and mid-infrared transmittance of the nanoPE film over 30 days, with insets showing dust accumulation on the film’s surface. **g** Demonstration of the cleaning effect of rain, with the inset displaying a contact angle of 134.5° for the nanoPE film. **h** Effect of UV radiation on the visible reflection of the nanoPE film on the bottom side. **i** Effect of UV radiation on the mid-infrared transmittance of the nanoPE film. **j** Average visible reflection and mid-infrared transmittance of the nanoPE film after UV exposure equivalent to 5 years, with insets showing the film’s surface. **k** UV reflection of the nanoPE film as well as the solar spectral irradiance in the UV band. Inset shows the chemical structure of PE

To validate the efficacy of the proposed radiative cooling method across a broad range of geographic regions, we conducted two additional experiments simulating extreme temperature conditions—specifically a hot environment at 39 °C and a cold environment at 5 °C (Fig. 6a). For these tests, we constructed a thermally insulated box with precise temperature control, achieved via circulated water, to replicate the extreme weather scenarios. The top of the box was sealed with a 10 μm-thick PE film, allowing the thermal radiation emitted by the LED light to escape while protecting the controlled internal environment from wind influences (further details can be found in Note S24, Fig. s30). As illustrated in Fig. 6b, the radiative cooling strategy we propose continues to demonstrate significant advantages in both hot and cold environments. In the hot environment, we observed a temperature decrease of 3.3 °C when the sky-facing cooling channel was active, indicating the effectiveness of the radiative cooling even under elevated ambient temperatures. Similarly, in the cold environment, a temperature reduction of 2.4 °C was achieved. The slightly reduced cooling effect in the cold environment can be attributed to the increased efficiency of non-radiative heat dissipation mechanisms, such as conduction and convection, which become more prominent at lower ambient temperatures. Furthermore, thermal imaging results confirm that the LED chip remains clearly visible through the single 360-µm-thick nanoPE film, underscoring the enhanced radiative cooling effect facilitated by the nanoPE cover. These findings suggest that the proposed sky-facing LED design, combined with the nanoPE film, can effectively manage heat in a wide range of environmental conditions, thereby expanding the potential applications of radiatively cooled LEDs to diverse climatic regions.

For outdoor applications, factors such as dust contamination, wind and rain exposure, and UV radiation are crucial.

#### Dust contamination (environmental pollution)

As illustrated in Fig. 6c, we exposed a 100-µm-thick nanoPE film, fabricated by hot-pressing, to outdoor conditions at the KAUST campus for 30 days to evaluate the impact of dust contamination on its spectral properties in both the visible (Fig. 6d) and MIR bands (Fig. 6e). The bottom side of the nanoPE film, which is designed to reflect light back to the ground, accumulated minimal dust, maintaining an average visible reflectance of 95% (black curve in Fig. 6f). However, dust accumulation notably affected the MIR transmittance, as indicated by the green curve in Fig. 6f, leading to a reduction in performance when dust was present. Fortunately, this dust can typically be removed by natural elements such as wind and rain, which helps restore the MIR transmittance.

#### Wind and rain exposure

As shown in Fig. 6f and the corresponding inset photos, the MIR transmittance increased from 0.62 on day 10 to 0.73 on day 15 as the wind cleared away the accumulated dust between two days. After 30 days, the MIR transmittance decreased from 0.81 to 0.68 but remained sufficient to allow most of the thermal radiation from the LED chip to escape into the sky. The nanoporous structure of the nanoPE film results in a high contact angle of 134.5° (see the inset of Fig. 6g and more details in Note S24, Fig. s31). This pronounced hydrophobicity suggests that the film effectively repels water, thereby potentially improving its stability in humid conditions. In Fig. 6g, an experiment simulating rain shows that droplets effectively clean the dust off the nanoPE film, restoring MIR transmittance to 0.81 (i.e., its original level, see pink dash line in Fig. 6e for spectral results), as detailed in the supplementary video showcasing this self-cleaning effect (see more discussion in Note S24).

#### UV radiation

Finally, we examined the impact of UV light on the spectral properties of the nanoPE film in both the visible (Fig. 6h) and mid-infrared (MIR) (Fig. 6i) bands. The 100 µm-thick nanoPE film underwent an accelerated UV aging test, as shown in the inset of Fig. 6h. This test involved exposing the film to strong UV light for 135 h under the irradiation of 2.5 kW/m^2^. Given that the average daily UV exposure in Saudi Arabia is around 200 W h/m^2^ ^64^, 133 h of UV exposure is equivalent to 5 years of outdoor exposure (following the estimation method reported by ref. ^65^). We measured the spectral properties at 27, 54, 81, and 135 h, respectively, corresponding to 1, 2, 3, and 5 years of equivalent outdoor exposure, respectively. As shown in Fig. 6h, the visible reflectance of the bottom side of the nanoPE film remained high (i.e., 95%, shown in Fig. 6j) over a 5-year period as the bottom side facing the ground is not illuminated by UV light. On the other hand, the nanoPE film exhibits great UV durability. This resilience is attributed to its molecular structure, which predominantly consists of C–C and C–H bonds with bond energies ranging from 300 to 600 kJ/mol (inset in Fig. 6k). These bonds are inherently resistant to UV-induced degradation, making polyethylene more durable than some other common polymers (e.g., Polystyrene, Polyurethane)^66,67^. Additionally, the nanoPE film’s strong UV reflectance, as shown in Fig. 6k, reduces UV light absorption, further enhancing its durability. However, UV exposure can still induce chain scission and cross-linking within the polyethylene (PE) backbone from the top surface, gradually degrading the material^66,67^. As illustrated in Fig. 6i, the film’s MIR transmittance decreases gradually with prolonged UV exposure. Despite this degradation, after five years, the MIR transmittance only dropped from 0.81 to 0.67 (Fig. 6j). This level of transmittance still permits the majority of thermal radiation from the LED chip to be emitted directly into the cold sky, demonstrating that the nanoPE film continues to effectively support radiative cooling even after extended UV exposure.

### Evaluation of energy savings

According to a DOE report, the total energy consumption by outdoor LED lights in the United States reached 76.9 TWh in 2018^68^. If the proposed sky-facing design were universally adopted, replacing all existing LED outdoor lights accordingly, the resulting energy savings would amount to a remarkable 1.9 TWh (see more discussion in Note S25). This substantial reduction in energy consumption would, in turn, lead to a noteworthy decrease of ~1.3 million metric tons (MMT) of CO_2_ emissions^69^. To put this into perspective, this reduction represents 0.03% of the total annual CO_2_ emissions by the US in 2018, which amounted to 4940 MMT^40^. The implications of such a reduction in energy consumption and carbon emissions are significant, signifying a promising potential for promoting energy efficiency and environmental sustainability.

## Discussion

In conclusion, our proposed approach achieves sky-facing radiative cooling for outdoor high-power LED streetlights. By utilizing a material with unique properties, specifically being infrared-transparent and visible-reflective like the nanoPE employed in this research, we have successfully harnessed the potential to release the heat generated by the LED chip through sky-facing radiative cooling while simultaneously reflecting the emitted light back to the ground for efficient illumination. The implementation of nanoPE as a radiative cooling material holds immense promise for outdoor lighting applications. It leverages its high visible reflectance to effectively reflect or diffuse visible light for proper illumination and utilizes its high thermal transparency to facilitate radiative cooling with the sky. Our comprehensive indoor and outdoor experiments have conclusively demonstrated that employing nanoPE as a cover for LED streetlights yields a substantial temperature reduction of 7.8 °C (with a cooling power of 1129 W/m^2^) in laboratory settings and 4.4 °C (with a cooling power of 1266 W/m^2^) in outdoor conditions, resulting in an impressive efficiency increase of approximately 4.9% and 4.4%, respectively. This notable efficiency improvement translates to substantial annual energy savings of 1.9 TWh in the United States, with a corresponding reduction of ~1.3 million metric tons of CO_2_ emissions. These significant contributions to energy conservation and carbon emission reduction underscore the practical viability of LED streetlights equipped with sky-facing capabilities, affirming their real-world application potential.

## Materials and methods

### Fabrication of nanoPE

To fabricate the nanoPE, high-density PE pellets (HDPE, melt index 2.2 g/10 min, Sigma-Aldrich) and ultrahigh molecular weight PE powder (UHMWPE, Alfa Aesar) are mixed in the paraffin oil (light, Fisher Chemical) at 180−200 °C for 30 min using an overhead stirrer. After sufficient stirring, a homogeneous polymer mixture is obtained, which can be further used to form the designed shapes by compression molding. During the forming process, the mixture is heated to an elevated temperature at which it softens and then cooled to room temperature after the desired shape is attained. After solidification, demixing of the homogeneous polymer solution occurs, which separates it into a polymer-rich solid phase and an oil-rich liquid phase due to thermally introduced phase separation. Finally, the paraffin oil in the film is extracted by methylene chloride (99.99%, Fisher Chemical), and an interconnected nanoporous polymer network is created.

### Measurements

For visible spectrum, reflection measurements were carried out using a spectrometer (Perkin Elmer Lambda 950) equipped with Tungsten-halogen and Deuterium lamps. Forward and backward scattered light was captured with a 150 mm visible/near-infrared integrating sphere coupled with a photomultiplier detector. For measurements in the mid-infrared portion of the spectrum, Fourier transform infrared spectrometer (Bruker HYPERION II), an integrating sphere (A562-G/Q) coated with diffuse gold reflectors and a DLaTGS detector were used.

## Supplementary information

Supporting Information

Supporting Video

## Data Availability

The authors declare that all data supporting the findings of this study can be found within the paper and its Supplementary information files. Additional data supporting the findings of this study are available from the corresponding author (Q.G.) upon reasonable request.
