# Supplementary material for: Sky cooling for LED streetlights
Source: Light Sci Appl. 2025 Feb 26;14:100. doi: 10.1038/s41377-024-01724-7 (PMC11861274; doi:10.1038/s41377-024-01724-7)
Supplement: Supplementary file 1 — Supporting Information [file 41377_2024_1724_MOESM1_ESM.docx]

***Supporting Information***

**Sky cooling for LED streetlights**

Saichao Dang^1‡^, Hasan H. Almahfoudh^1‡^, Abdulrahman M. Alajlan^2^, Hussam Qasem^2^, Jiake Wang^1^, Yingkun Zhu^1^, Osman M. Bakr^3^, Boon Ooi^4^, Qiaoqiang Gan^1^*

1.Sustainable Photonics Energy Research Laboratory, Material Science Engineering, PSE, King Abdullah University of Science and Technology (KAUST), Thuwal 23955-6900, Saudi Arabia

2.Future Energy Technology Institute, King Abdulaziz City for Science and Technology, Riyadh 11442, Saudi Arabia

3. Functional Nanomaterials Laboratory, Material Science and Engineering, PSE, KAUST, Thuwal 23955-6900, Saudi Arabia

4. Photonics Laboratory, Electrical and Computer Engineering, CEMSE, KAUST, Thuwal 23955-6900, Saudi Arabia

‡ These authors contributed equally to this work.

*Correspondence: [qiaoqiang.gan@kaust.eu.sa](mailto:qiaoqiang.gan@kaust.eu.sa)

**Table of Contents:**

**Note S1**: Radiative heat flux between an object and the cold sky

**Note S2**: Detailed calculation process for the cooling power of the sky-facing radiative cooling channel when open or closed

**Note S3:** Radiative cooling power for commercial LED streetlight

**Note S4:** Classic scattering theory

**Note S5**: Influence of thermal conductivity

**Note S6**: Biaxially oriented PE (BOPE) in the dry process

**Note S7**: BOPE in the wet process

**Note S8**: Pore size distribution of nanoPE film

**Note S9**: Impact of thickness on the optical properties

**Note S10**: Tensile test of nanoPE film

**Note S11**: Influence of gap between the chip and the cover

**Note S12**: Detailed experimental settings

**Note S13**: Characterization of junction temperature

**Note S14**: Characterization of emitted light (luminous flux, CCT)

**Note S15:** The results of spectrometer

**Note S16**: Examination of the spectral characteristics determined through optical fiber measurements

**Note S17**: Weather data on the days of outdoor experiment under clean sky and under cloudy sky

**Note S18**: Radiative cooling effect on a commercial LED lamp

**Note S19**: Characterization of the LED light under cloudy sky

**Note S20**: Cooling power calculation for the indoor and outdoor experiments

**Note S21:** Visible transmission of nanoPE

**Note S22**: Relationship between luminosity and chip surface temperature

**Note S23**: Effect on the distribution of light intensity

**Note S24**: More details about the experiment under extreme environments and practical conditions

**Note S25**: More discussion about the energy saving estimation

**List of Figures:**

**Figure S1** Schematic of radiative heat transfer between an object and the atmosphere.

**Figure S2** The applied atmospheric transmittance.

**Figure S3** Scheme of the sky-facing radiative cooling channel open (a) or closed (b).

**Figure S4** Heat transfer analysis of sky-facing radiative cooling of LED chip. a. Schematic of heat transfer process of LED chip with direct sky-facing radiative cooling. b. The heat transfer network of LED chip with thermal opaque cover or thermal transparent cover.

**Figure S5** Practical LED streetlight and different configurations. (a) The practical LED streetlight in Kaust. (b) The corresponding structure of the streetlight and the configuration of ground-facing LED light with glass cover. (c) The configuration of sky-facing LED light with glass cover. (d) The configuration of sky-facing LED light with nanoPE cover.

**Figure S6** The schematic geometry model of the LED light in software. Purple: LED chip; Yellow: substrate. Green: front cover. Gray: four walls.

**Figure S7** The LED chip temperature as a function of the thermal conductivity of the nanoPE film.

**Figure S8** The traditional BOPE process. The applied biaxially oriented film stretching machine (a) with zoom-up clamp (b).

**Figure S9** The SEM of BOPE film in dry process.

**Figure S10** Schematic of BOPE in wet process.

**Figure S11** Characterization and result of pore size distribution of nanoPE film. a. SEM result. b. Statistical analysis of the pore size distribution. c. Pore size distribution based on BET measurement.

**Figure S12** (a) The spectral visible reflection. The spectral intensity of the LED light is represented by blue dash line. (b) Averaged reflection as a function of the thickness. (c) The spectral MIR transmission.

**Figure S13** The images of tensile test of nanoPE with 15 μm, 30 μm and 60 μm.

**Figure S14** One commercial LED product with PVC cover.

**Figure S15** The sustained force of nanoPE with different thicknesses.

**Figure S16** The influence of the gap between the LED chip and the cover. (a) Schematic illustration of the experimental setup. (b) Chip temperature as a function of the gap distance. (c) A photograph of the experimental setup. (d) The illuminated area of illuminance higher than 10 lux as a function of the gap distance.

**Figure S17** The detailed indoor setup to prevent the cooling effect by evaporated liquid nitrogen.

**Figure S18** The thermal emissivity of the front-side of LED chip (inset).

**Figure S19** Junction temperature (Tj) characterization and result. (a) The setup for the measurement of optical and electrical properties of the applied LED chip. (b) The forward voltage as a function of Tj under the current of 0.2 A.

**Figure S20** (a) The luminous properties of the applied LED chip under 0.2 A and different junction temperatures. (b) The forward voltage as a function of junction temperature.

**Figure S21** (a) The outdoor experiment demonstration under an ambient temperature of ~21 ℃. (b) The temperature of the LED chip under cooling off and cooling on. (c) The spectral properties of the emitted light under conditions of “cooling on” and “cooling off”. (d) Zoom-in view of the blue peak.

**Figure S22** The outdoor temperature and wind speed during the operating of outdoor experimental test under clean sky.

**Figure S23** The outdoor temperature and wind speed during the operating of outdoor experimental test under cloudy sky.

**Figure S24** (a) The experiment demonstration of the commercial LED product with original cover, cooling-off cover and cooling-on cover. (b) The spectral properties of the emitted light with three covers. (c) The chip surface temperature of this LED product. (d) The illuminated area of illuminance higher than 10 lux.

**Figure S25** The spectral intensity of the emitted light from LED chip under clean sky.

**Figure S26** Voltage and temperature vs peak wavelength at cooling on and cooling off under cloudy sky.

**Figure S27** The spectral visible transmission.

**Figure S28** The characterized luminous flux (lumens) using an integrating sphere (inset) as the function of the surface temperature of the LED chip.

**Figure S29** Illuminance as a function of horizontal distance from the LED chip.

**Figure S30** Outdoor experiment under extreme environments.

**Figure S31** The characterization of contact angle.

**List of Tables:**

**Table S1** Stable temperatures and cooling powers of practical LED streetlights: Ground-facing configuration with normal transparent (glass) front cover vs. Sky-facing configuration with glass cover and nanoPE cover.

**Table S2** The averaged thermal emissivity of each component in the LED streetlight.

**Table S3** The material parameters of each part of the customized LED light.

**Note S1: Radiative heat flux between an object and the cold sky**

According to the Planck’s law^[R1]^, the thermal radiation from an object can be expressed as

$P_{\text{rad}}\left(T\right)=A\int I_{\text{BB}}\left(T,\lambda\right)\varepsilon\left(\lambda,\theta\right)\text{d}\lambda\int_{0}^{\text{π}/2} 2\text{π}\cos\theta\sin\theta\text{d}\theta$ (S1)

where $\int\text{d}\Omega\text{=2π}\int_{0}^{\text{π}/2} \sin\theta\text{d}\theta$ is the angular integral over a hemisphere. $I_{\text{BB}}\left(T,\lambda\right)=\frac{2\text{hc}^{2}}{\lambda^{5}}\frac{1}{\text{e}^{\text{hc}/\left(\lambda\text{k}_{\text{B}}T\right)}-1}$ is the spectral radiance of a blackbody at temperature *T*, where h is Planck’s constant, k_B_ is the Boltzmann constant, c is the speed of light in vacuum, *A* is area. $\varepsilon\left(\lambda,\theta\right)$ is the wavelength and angular dependent emissivity of the object (i.e., 1 for a blackbody). In this work, the considered spectral range is 4~40 *μ*m.


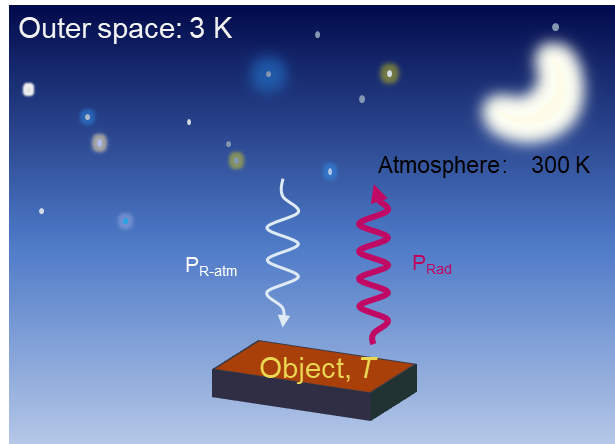


Figure S Schematic of radiative heat transfer between an object and the atmosphere.

The schematic of radiative heat transfer between an object and the sky is shown in Fig. S1. Based on the energy balance law and the radiative heat transfer process shown in Fig. S1, the corresponding net radiative heat flux (i.e., *P*_f_) can be calculated by

$P_{\text{f}}=P_{\text{rad}}\left(T\right)-P_{\text{amb}}\left(T_{\text{amb}}\right)$ (S2)

*P*_rad_ is the radiated power of the object, which can be obtained by Eq. (S1). *P*_amb_ is absorbed power from the incident ambient thermal radiation. The ambient refers to the atmosphere for sky-facing configuration. The absorbed radiative heat of object from the atmosphere can be calculated by

$P_{\text{amb}}\left(T_{\text{amb}}\right)=A\int\int I_{\text{BB}}\left(T_{\text{amb}},\lambda\right)\alpha\left(\lambda,\theta\right)\varepsilon_{\text{amb}}\left(\lambda,\theta\right)\text{d}\lambda\cos\theta\text{d}\Omega$ (S3)

where *T*_amb_ is the temperature of the atmosphere. $\alpha\left(\lambda,\theta\right)$ is the wavelength and angular dependent absorbance of the object, which equals $\varepsilon\left(\lambda,\theta\right)$ according to the Kirchoff’s law^[R1]^ ($\alpha\left(\lambda,\theta\right)\text{=}\varepsilon\left(\lambda,\theta\right)$). The angle dependent emittance of the atmosphere is given by

$\varepsilon_{\text{atm}}\left(\lambda,\theta\right)=1-t\left(\lambda\right)^{1/\cos\theta}$ (S4)

where *t*(*λ*) is the atmosphere transmittance in the normal direction and *θ* is the zenith angle ^[R2]^. The atmospheric transmittance varies with the weather conditions and regions. In this work, we used the atmospheric transmittance shown in Fig. S2 ^[R3]^.

Figure S The applied atmospheric transmittance.

**Note S2: Detailed calculation process for the cooling power of the sky-facing radiative cooling channel when open or closed**

Fig. S3a and Fig. S3b show the schematics of the channel of sky-facing radiative cooling is open or closed, respectively.

**Cooling On:** Considering the non-radiative cooling term, the radiative cooling power of sky-facing can be calculated by

$P_{\text{cool-s}}=P_{\text{rad}}\left(T\right)-P_{\text{amb}}\left(T_{\text{amb}}\right)-P_{\text{c}}$ (S5)

where *P*_rad_ and *P*_amb_ can be found in Eq. (S1) and Eq. (S3). *P*_c_ is the heat loss of the object due to the combination of the convection and conduction heat transfer between the object and the ambient air, which can be calculated by

$P_{\text{c}}=h_{\text{com}}A\left(T-T_{\text{amb}}\right)$ (S6)

with the air temperature (*T*_amb_) being 300 K and *h*_com_ being the non-radiative heat transfer coefficient (assuming natural convection with *h*_com_=6.9 W/(m^2^∙K) in this work ^[R4]^).

**Cooling Off:** The radiative cooling power with the channel closed can be obtained by a similar process. In this process, the infrared transmission of the atmospheric window is not required as the thermal opaque cover at 300 K blocks the channel share the same temperature. Taking the conduction and convection heating loss into account as shown in Fig. S3b, the cooling power of the ground-facing radiative cooling can be calculated by

$P_{\text{cool-g}}=P_{\text{rad}}\left(T\right)-P_{\text{o}}\left(T_{\text{o}}\right)-P_{\text{c}}$ (S7)

where *P*_rad_ can be obtained by Eq. (S2). *P*_o_ (*T*_o_) is the absorbed thermal radiation by the object from the thermal opaque cover, which can be calculated by Eq. (S1) with *T*_o_=300 K. The heat loss of the object due to convection and conduction heat transfer, i.e., *P*_c_ can be calculated by Eq. (S6). According to the above process, the cooling power of cooling channel open or closed can be obtained accordingly.


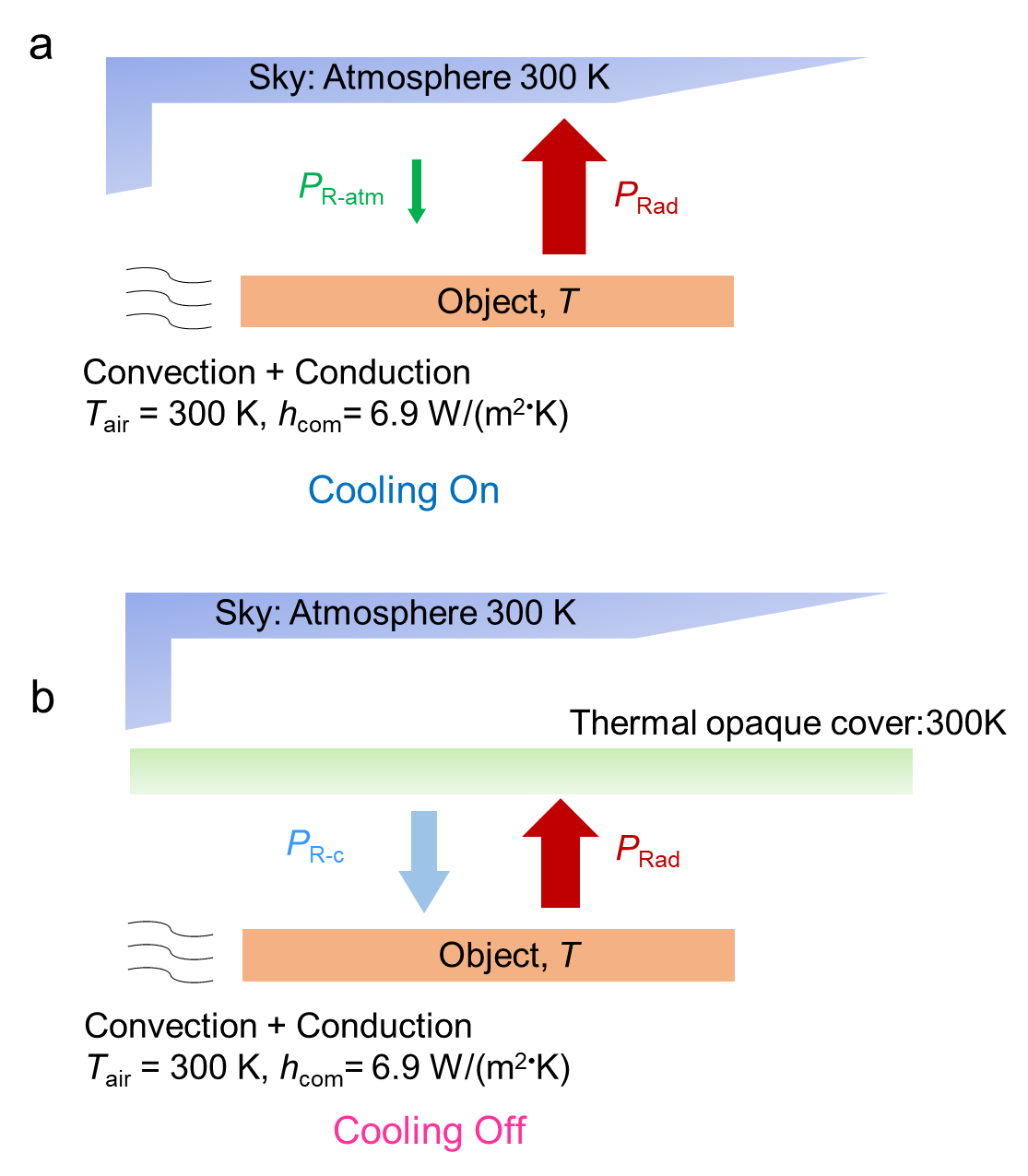


Figure S Scheme of the sky-facing radiative cooling channel open (a) or closed (b).

**Heat transfer networks**

To estimate the cooling potential of sky-facing LED applications, we further analysis the heat transfer process between the chip and sky according to the schematic shown in Fig. S4a. For a better understanding, the heat transfer networks for LED chip with thermal opaque or thermal transparent covers are provided, as shown in Fig. S4b.

For a LED chip with a thermal opaque cover with a temperature *T*_c_ (e.g., 300 K in this discussion), the cooling power from radiative and non-radiative heat transfer can be determined by

$P_{\text{c-R}}\left(T\right)=A\left(\varepsilon\sigma\left(T^{4}-T_{\text{c}}^{4}\right)\right)$ (S8)

and

$P_{\text{c-nonR}}\left(T\right)=Ah_{\text{h-c}}\left(T-T_{\text{air}}\right)$ (S9)

with the emissivity of the cover (i.e., *ε*_c_) being 1 and the non-radiative heat transfer coefficient (i.e. *h*_h-c_) being 6.9 W/(m^2^∙K). The above radiation heat transfer process can be evaluated by the corresponding heat transfer coefficient *h*_r-c_ (Fig. S4b), which can be obtained by the corresponding cooling power and temperature difference:

$P_{\text{c-R}}\left(T\right)=Ah_{\text{r-c}}\left(T-T_{\text{amb}}\right)$ (S10)

With a thermal transparent cover, the thermal radiation from the chip can be released to the sky directly, as shown by the red dash square in Fig. S4b. Therefore, the corresponding cooling power by radiation can be calculated by

$P_{\text{c-R}}\left(T\right)=A\tau_{\text{c}}\left(\varepsilon\sigma T^{4}-P_{\text{amb}}\left(T_{\text{amb}}\right)\right)-A\left(\varepsilon_{\text{c}}\sigma T_{\text{c}}^{4}+\text{r}_{\text{c}}\varepsilon\sigma T^{4}\right)$ (S11)

The thermal reflectance, emittance and the transmittance of this cover with r_c_+ε_c_+τ_c_=1 in this calculation is assumed to be r_c_=0, ε_c_=0 and τ_c_=1, respectively. The radiation from the ambient (i.e., *P*_amb_(*T*_amb_)) can be obtained from Eq. (S3) with *T*_amb_=300 K. Under cloudy sky, we used half the atmospheric transmittance for convenience. The cooling power by non-radiation is also determined by Eq. (S9).

According to the above calculating process and the given parameters, the cooling power and comprehensive radiative and non-radiative heat transfer coefficient can be determined.


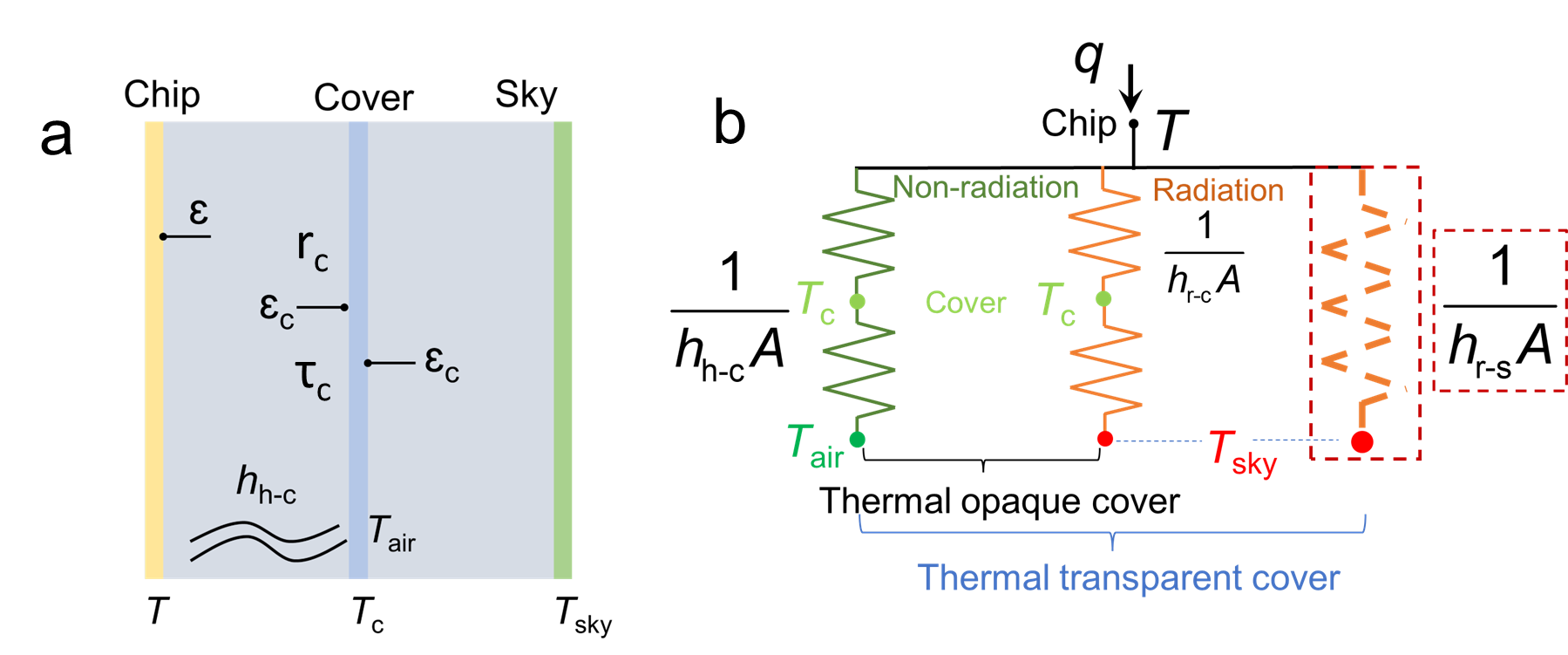


Figure S4 Heat transfer analysis of sky-facing radiative cooling of LED chip. a. Schematic of heat transfer process of LED chip with direct sky-facing radiative cooling. b. The heat transfer network of LED chip with thermal opaque cover or thermal transparent cover. For a thermally opaque cover, heat is transferred from the chip to the cover through both non-radiative and radiative processes, characterized by the coefficients *h*_h−c_​ and *h*_r_*_−_*_c_, respectively. In contrast, a thermally transparent cover opens an additional radiative cooling channel from the chip to the sky, with the coefficient *h*_r−s​_.

**Note S3: Radiative cooling power for commercial LED streetlight**

To elucidate the logic behind the enhanced cooling potential of our proposed sky-facing architecture, we begin with an analysis of a commercial ground-facing LED streetlight installed on the KAUST campus, as shown in Fig. S5a. As detailed in Fig. S5b, this LED streetlight features a front glass cover, a central LED light array, and a robust thermal management package with the backside oriented skyward. Temperature measurements of these components using a probe revealed temperatures of 71.7 °C for the backside, 95.5 °C for the central LED panel, and 47.2 °C for the front glass cover, as recorded in Table S1. Rereferring Note S2, a non-radiative heat transfer coefficient of *h*_com_ is required, which is 11.3 W/(m^2^∙K) in these outdoor experiments (*h*_com_=8.3+2.5*V*_w_ with an average wind speed of 1.2 m/s and an ambient temperature of 27 °C) ^[R4]^. Utilizing the known thermal emissivities of the backside package and front glass cover (data presented in Table S2), we calculated the total cooling power to be 1140 W/m². However, by reversing the orientation of the LED fixture to position the front glass skyward (illustrated in Fig. S5c), the cooling power measured was slightly lower at 1124 W/m² due to the cooler front cover facing the sky and the warmer backside facing the ground. This configuration, while reducing cooling power, would unfortunately direct all visible light skyward, contravening practical engineering requirements for LED streetlights. Our original intention was to maintain ground-directed illumination. Thus, as illustrated by Fig. S5d, by fully opening the radiative cooling channel (i.e. 100% thermal transmission), the hottest LED panel will face the sky directly. As a result, the theoretical upper limit for the cooling potential is 1650 W/m². Considering a thermal transmission of ~80%, the overall cooling power is 1522 W/m^2^, still 382 W/m^2^ higher than the original position in Fig. S5b. This envelope estimation revealed the potential of the sky-facing radiative cooling channel and revealed the promise to further optimize the spectral selectivity of the nanoPE film.


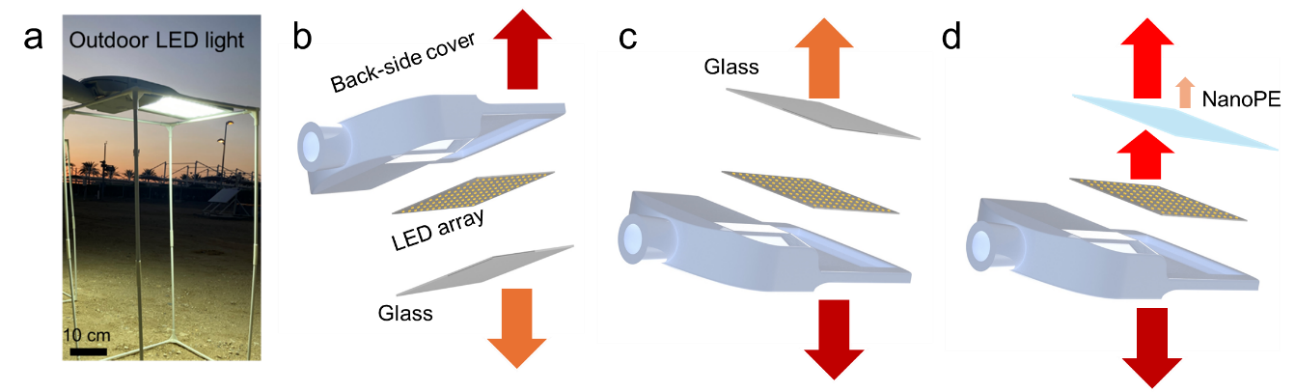


Figure S5 Practical LED streetlight and different configurations. (a) The practical LED streetlight in Kaust. (b) The corresponding structure of the streetlight and the configuration of ground-facing LED light with glass cover. (c) The configuration of sky-facing LED light with glass cover. (d) The configuration of sky-facing LED light with nanoPE cover.

Table S1 Stable temperatures and cooling powers of practical LED streetlights: Ground-facing configuration with normal transparent (glass) front cover vs. Sky-facing configuration with glass cover and nanoPE cover.

|  | Chip (*T*_c_) | Net cooling power (W/m^2^) | Back-side cover | Net cooling power (W/m^2^) | Front-side cover | Net cooling power (W/m^2^) | Total  (W/m^2^) |
| --- | --- | --- | --- | --- | --- | --- | --- |
| Ground-facing | 95.5 ℃ |  | 71.7 ℃ | 902  **(sky facing)** | 47.2 ℃ | 238  **(ground facing)** | 1140 |
| Sky-facing with glass | 95.8 ℃ |  | 72.5 ℃ | 782  **(ground facing)** | 44.1 ℃ | 342  **(sky facing)** | 1124 |
| Sky-facing with nanoPE | 92.3 ℃ | 641 (100%)  513 (80%)  352 (55%)  **(sky facing)** | 71.2 ℃ | 751  **(ground facing)** | 46.4 ℃ | 258  **(sky facing)** | 1650 (100%)  1522 (80%)  1361 (55%) |

Table S2 The averaged thermal emissivity of each component in the LED streetlight.

| **Component** | **Emissivity** |
| --- | --- |
| Chip | 0.95 |
| Back-side cover | 0.93 |
| Front-side glass cover | 0.93 |
| NanoPE | 0.15 |

**Note S4: Classic scattering theory**

The scattering effects of particles for different wavelengths can be described using classic scattering theories, including Mie scattering theory and Rayleigh scattering theory ^[R5]^.

Mie scattering theory describes the scattering of electromagnetic waves by spherical particles that are comparable in size to the wavelength of the light. This theory is a solution to Maxwell's equations for the scattering of light by a spherical particle. Mie theory is particularly useful for understanding scattering in cases where the particle size is similar to or larger than the wavelength of the incident light. The scattering efficiency is given by the following series:

$Q_{\text{sca}}=\frac{2}{x^{2}}\sum_{n=1}^{\infty} \left(2n+1\right)\left(\left|a_{n}\right|^{2}+\left|b_{n}\right|^{2}\right)$ (S12)

where $x=\frac{2\pi r}{\lambda}$ is the size parameter, 𝑟 is the radius of the spherical particle, λ is the wavelength of the incident light, *a*_n_ and *bn*  are the Mie coefficients that depend on the size parameter 𝑥, the relative refractive index $\frac{m}{m_{0}}$ and the wavelength.

Rayleigh scattering theory describes the scattering of light by particles that are much smaller than the wavelength of the light. This type of scattering is highly wavelength-dependent and is responsible for phenomena like the blue color of the sky and the red color of sunsets.

In conclusion, Mie scattering applies to particles that are comparable in size to or larger than the wavelength of light. It utilizes the size parameter *x* and Mie coefficients *a*_n_ and *bn* to describe the scattering behavior. Rayleigh scattering, on the other hand, is used for particles much smaller than the wavelength of light. It scales with 1/λ^4^, leading to wavelength-dependent phenomena such as the color of the sky. In our calculations, the refractive index of polyethylene (PE) is 1.51 in the visible band ^[R6]^ and 1.53 in the mid-infrared band ^[R7]^.

**Note S5: Influence of thermal conductivity**

To comprehensively explore how the thermal conductivity of the cover impacts the LED chip's temperature performance, we conducted a thermal simulation using Multiphysics COMSOL. The geometry model is obtained based on the customized LED light. This schematic geometry model in the software and the detailed components are shown in Fig. S6. This light is placed in an environment with a temperature of 20 ℃. In order to make the simulation analysis proceed effectively, the structures of multilayer quantum layer, gold wires, and bonding pads in LED are neglected. It is also assumed that 80% of the input power (i.e., 5.2 W) is converted to heat. Table S3 shows material parameters of each part of LED lamp. In the simulations, three heat transfer modes, i.e., conduction, convection and radiation, are considered. The conduction heat transfer is considered within these solid components. The laminar flow convection of the air inside the shield is considered. Each exposed surface with the specific emissivity is considered to evaluate the radiation heat transfer. To simulate the thermal transmission of nanoPE, we used the semi-transparent surface settings in the software with a transmittance of 0.9 within the mid-infrared band. To demonstrate the junction temperature, we used the highest temperature within the chip as the indicator.


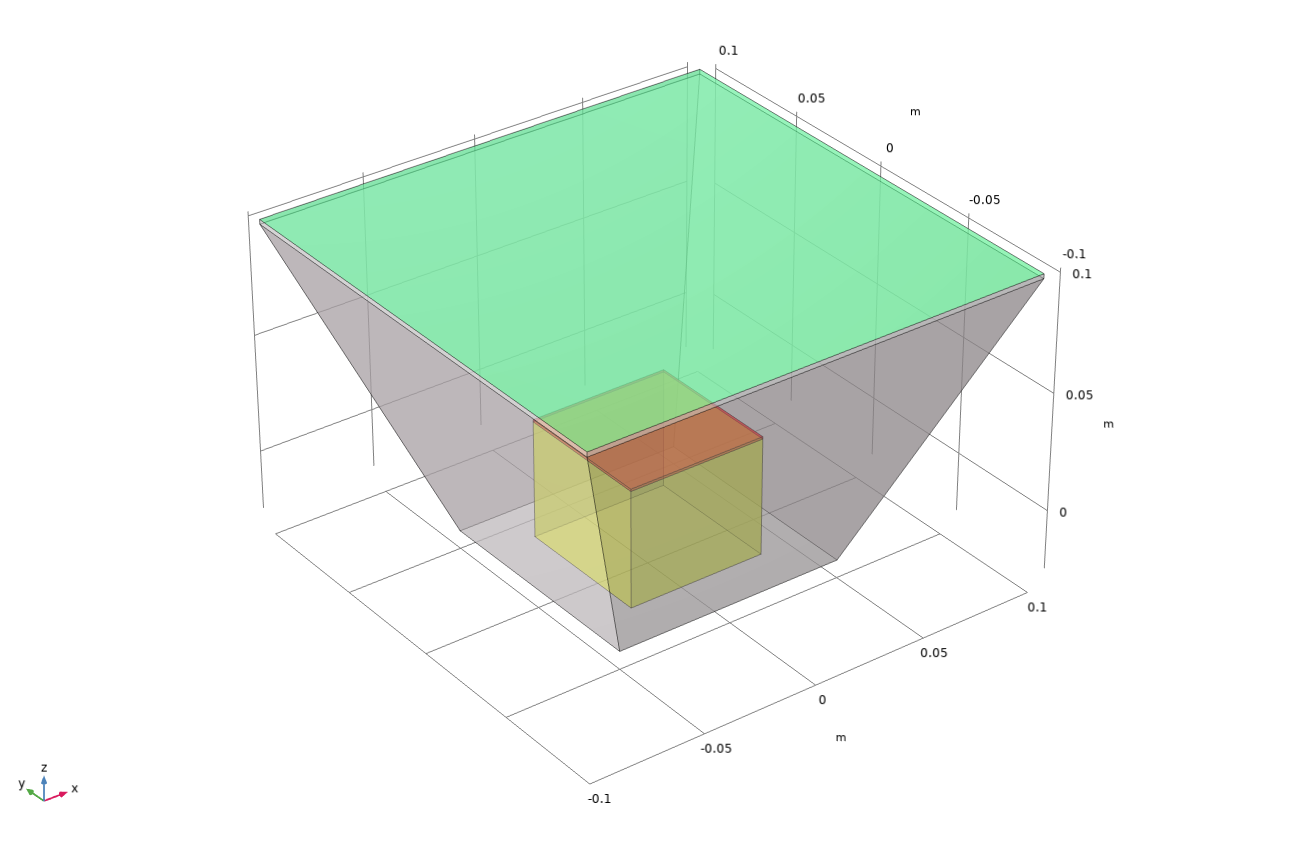


Figure S The schematic geometry model of the LED light in software. Purple: LED chip; Yellow: substrate. Green: front cover. Gray: four walls.

Table S3 The material parameters of each part of the customized LED light.

| Component | Material | Dimension (mm) | Density (kg/m^3^) | Thermal conductivity (W/(m∙k)) | Thermal capacity (J/(kg∙K)) | Thermal emissivity |
| --- | --- | --- | --- | --- | --- | --- |
| Chip | PCB | 50×50×0.2 | 2970 | 178 | 700 | 0.91 |
| Substrate | PMMA | 60×60×50 | 1202 | 237 | 903 | 0.92 |
| Four walls | PMMA | Hight=110, Thickness=2 | 1185 | 0.20 | 1500 | 0.93 |
| Front cover | PE | 200×200×0.1 | 1200 | 0.2-30 | 1200 | 0.05 |

Throughout the simulation, the front PE cover retained a consistent thickness of 100 μm with unchanged spectral properties, while the thermal conductivities varied from 0.2 W/(m·K) to 30 W/(m·K). As illustrated in Fig. S7, the LED chip's temperature, representing its maximum value, remains constant despite higher thermal conductivity. A rise from 0.2 W/(m·K) to 30 W/(m·K) only results in a 0.08 ℃ drop in LED temperature. These simulated findings indicate that the nanoPE film's thermal conductivity has a negligible effect.

Although the effect of the nanoPE film's thermal conductivity is insignificant, we examined its thermal properties before and after stretching to address the reviewer's concerns regarding the PE film's thermal conductivity impact by hotdisk method (TPS 2500 S). Our findings revealed a shift in thermal conductivity from 0.2 W/(m·K) to 0.9 W/(m·K) after the stretching process in our study.

Figure S7 The LED chip temperature as a function of the thermal conductivity of the nanoPE film.

**Note S6: Biaxially oriented PE (BOPE) in the dry process**


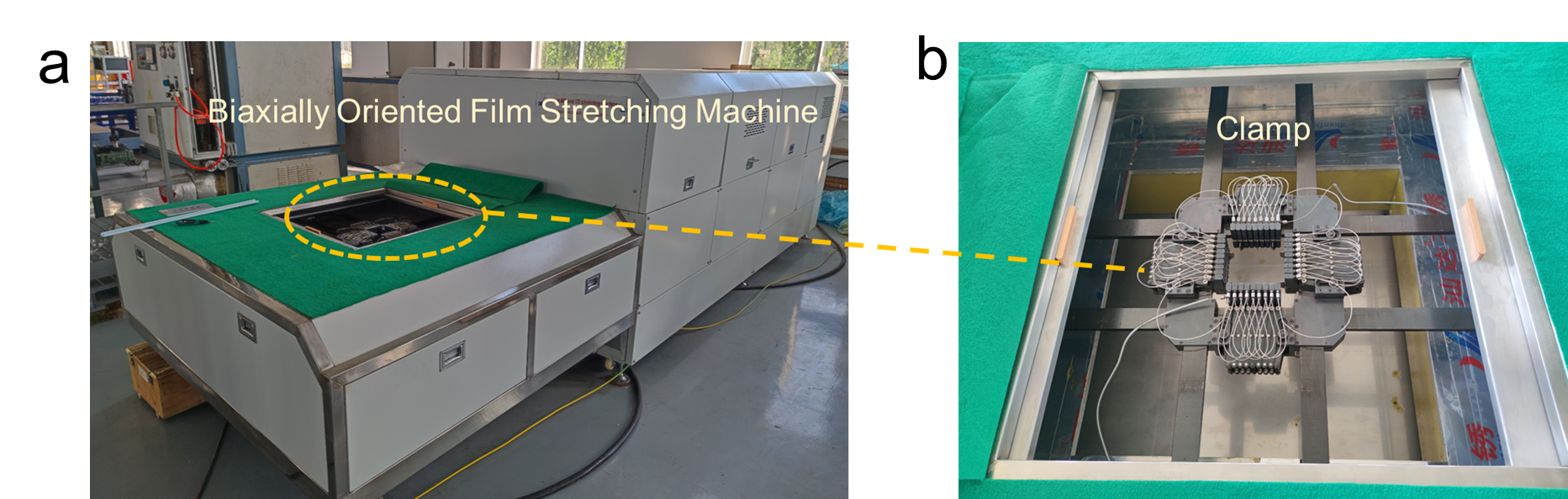


Figure S The traditional BOPE process. The applied biaxially oriented film stretching machine (a) with zoom-up clamp (b).

To realize BOPE in dry process (Fig. S8), we firstly preheat the biaxial stretching machine for 3 minutes, and then perform simultaneous biaxial stretching at a rate of 40 mm/s to achieve a 2.74x stretch ratio within a chamber at 145 ℃. In this study, we utilized polyethylene (PE) with a molecular weight of approximately 600,000 to ensure that the resulting PE film exhibits robust mechanical properties.

As shown in Fig. S9, the BOPE in dry process introduced cracks (white square) and nano pores (green square) into the film.


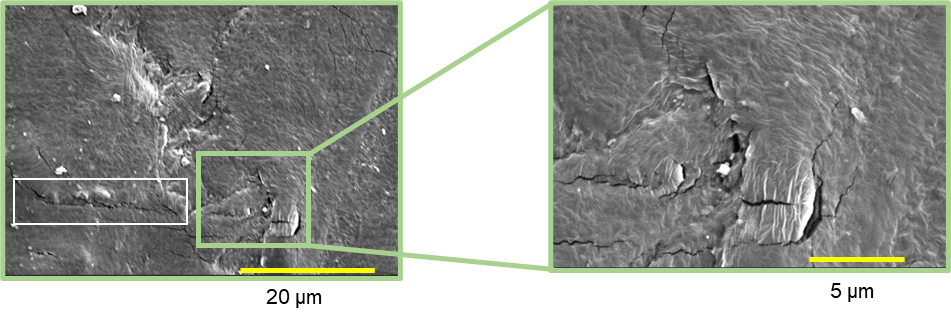


Figure S The SEM of BOPE film in dry process.

**Note S7: BOPE in the wet process**

Fig. S10 illustrates the schematic of the wet BOPE (Biaxially Oriented Polyethylene) process with extracting nethod used to fabricate nano PE. Initially, PE with a molecular weight of approximately 2 M is blended with paraffin oil and then extruded. The mixed PE film is subjected to biaxial orientation within a heat chamber at around 110 ℃ to create nanopores. Subsequently, methylene chloride is utilized to extract the paraffin oil and generate the nanostructure. To reduce production costs, methylene chloride is recycled. Finally, a heat setting process is applied to form a stable nano PE film.


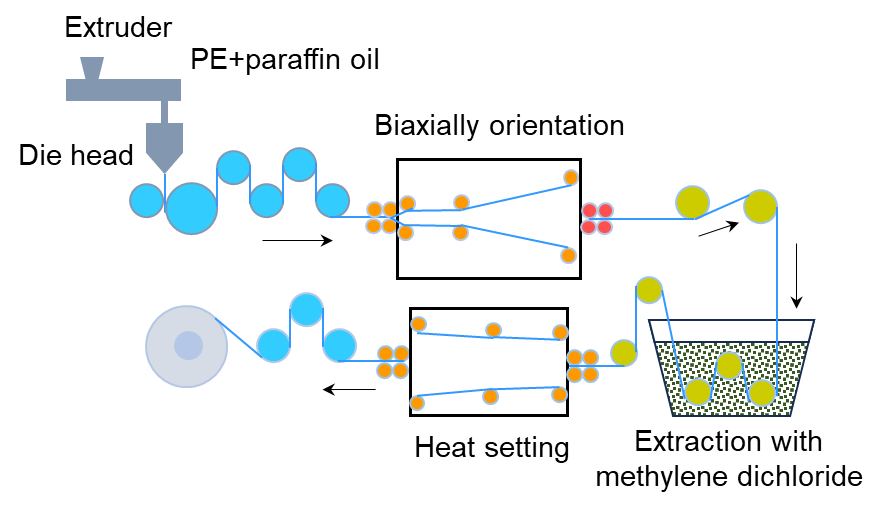


Figure S Schematic of BOPE in wet process.

**Note S8: Pore size distribution of nanoPE film**

We analyzed the dimensions of pores that are visible on the surface using its SEM image, as shown in Fig. S11a. Employing image analysis software (ImageJ), we directly measured the size of each pore in the SEM image and performed statistical analysis on their distribution, as illustrated in Fig. S11b. The analysis revealed a predominant distribution of pores ranging from 20 to 120 nm, with a peak concentration at approximately 100 nm.

To gain a comprehensive insight into the pore size distribution throughout the volume of the nanoPE film, we utilized a commercial system (Micromeritics® TriStar II) known for its expertise in analyzing pore size distribution, particularly in membranes, based on the Brunauer-Emmett-Teller (BET) theory^[R8]^. Fig. S11c demonstrates that our nanoPE film exhibits a prevalent pore distribution spanning 30-120 nm, with a peak concentration at 80 nm, significantly contributing to the scattering of visible light. The consistency between both sets of measurements affirms the reliability of our data.


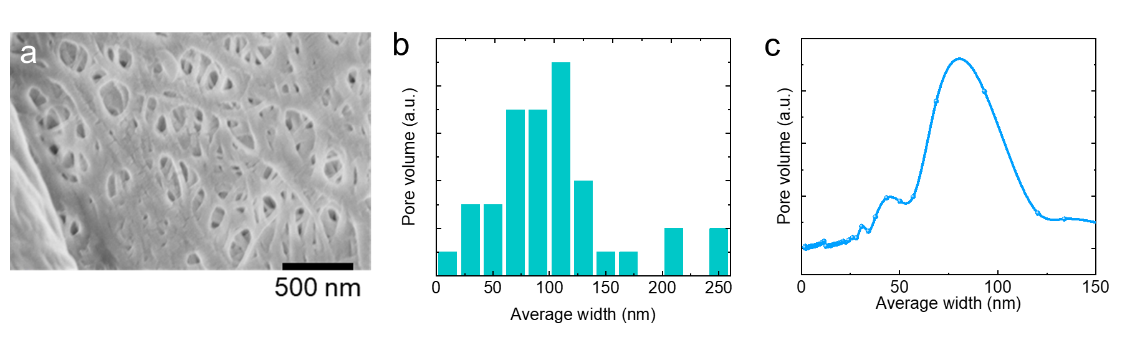


Figure S11 Characterization and result of pore size distribution of nanoPE film. a. SEM result. b. Statistical analysis of the pore size distribution. c. Pore size distribution based on BET measurement.

**Note S9:** **Impact of thickness on the optical properties**

For the proposed nanoPE cover, we characterized the reflection spectrum of the nanoPE film as a function of the film thickness, as shown in Fig. S12a. By employing the measured spectral intensity of the LED light (i.e., *I*(λ), represented by the blue dashed line in Fig. S12a) using Eq. (S12), one can estimate the averaged visible reflection of these nanoPE films with various thicknesses:

$\tilde{R}_{\text{e}}=\frac{\int_{0.4\mu\text{m}}^{0.8\mu\text{m}} I\left(\lambda\right)\cdot R_{\text{e}}\left(\lambda\right)d\lambda}{\int_{0.4\mu\text{m}}^{0.8\mu\text{m}} I\left(\lambda\right)d\lambda}$ (S13)

As shown in Fig. S12b, the averaged visible reflection of the nanoPE film can be enhanced to >90% with the thickness over 60 μm. Remarkably, one still can obtain reasonably high transmission in mid IR spectral range to enable radiative cooling to the LED chip. By considering the spectral mid-infrared transmission data shown in Fig. S12c, the averaged mid-IR transmission reaches 86% with the thickness 60 μm, allowing meaningful cooling powers to the LED chip.


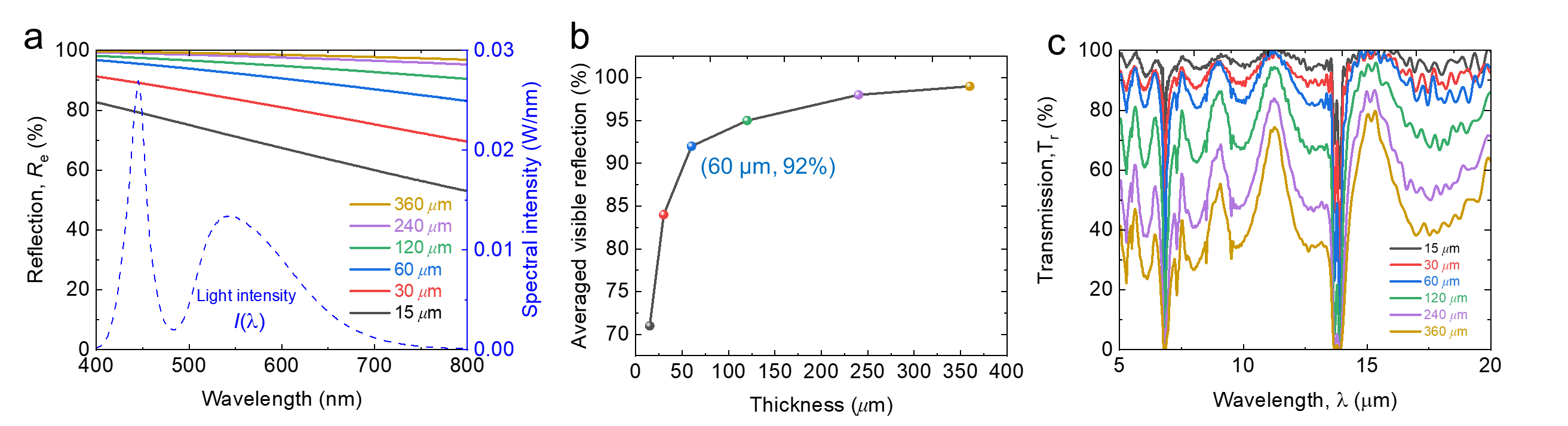


Figure S12. (a) The spectral visible reflection. The spectral intensity of the LED light is represented by blue dash line. (b) Averaged reflection as a function of the thickness. (c) The spectral MIR transmission.

**Note S10: Tensile test of nanoPE film**

We tested the tensile strength of the nanoPE film by a material testing machine (INSTRON/5969). For the tensile strength test, the sample was 2 cm wide and 4 cm long, and the gauge distance was 1 cm long. The displacement rate was 10 mm/min. The tensile test of nanoPE with more layers are shown in Fig. S13. This tensile strength significantly exceeds that of conventional materials traditionally employed in commercial outdoor LED lighting packages, such as PVC, which is known to exhibit a tensile strength of approximately 15 MPa (e.g. [R9, R10]). Consequently, it is estimated that a nanoPE film with a thickness of 360 μm provides tensile protection comparable to PVC films of roughly 1.9 mm thickness (i.e. 81MPa*0.36mm/15MPa=1.9 mm), which is considered sufficient for use in commercial applications. For instance, as illustrated in Fig. S14, a commercial LED product ^[R11]^ employed a PVC cover with the thickness of 0.65 mm only [i.e., (outer width 12.3 mm – inner width 11 mm)/2, sourced from a commercial product manual]. Furthermore, increasing the thickness of the nanoPE film enhances its ability to endure even greater forces, as shown in Fig. S15. However, for commercial viability, additional attributes such as waterproofing, dust resistance, and windproofing ^[R12]^ are essential, extending beyond mere radiative cooling performance.

**
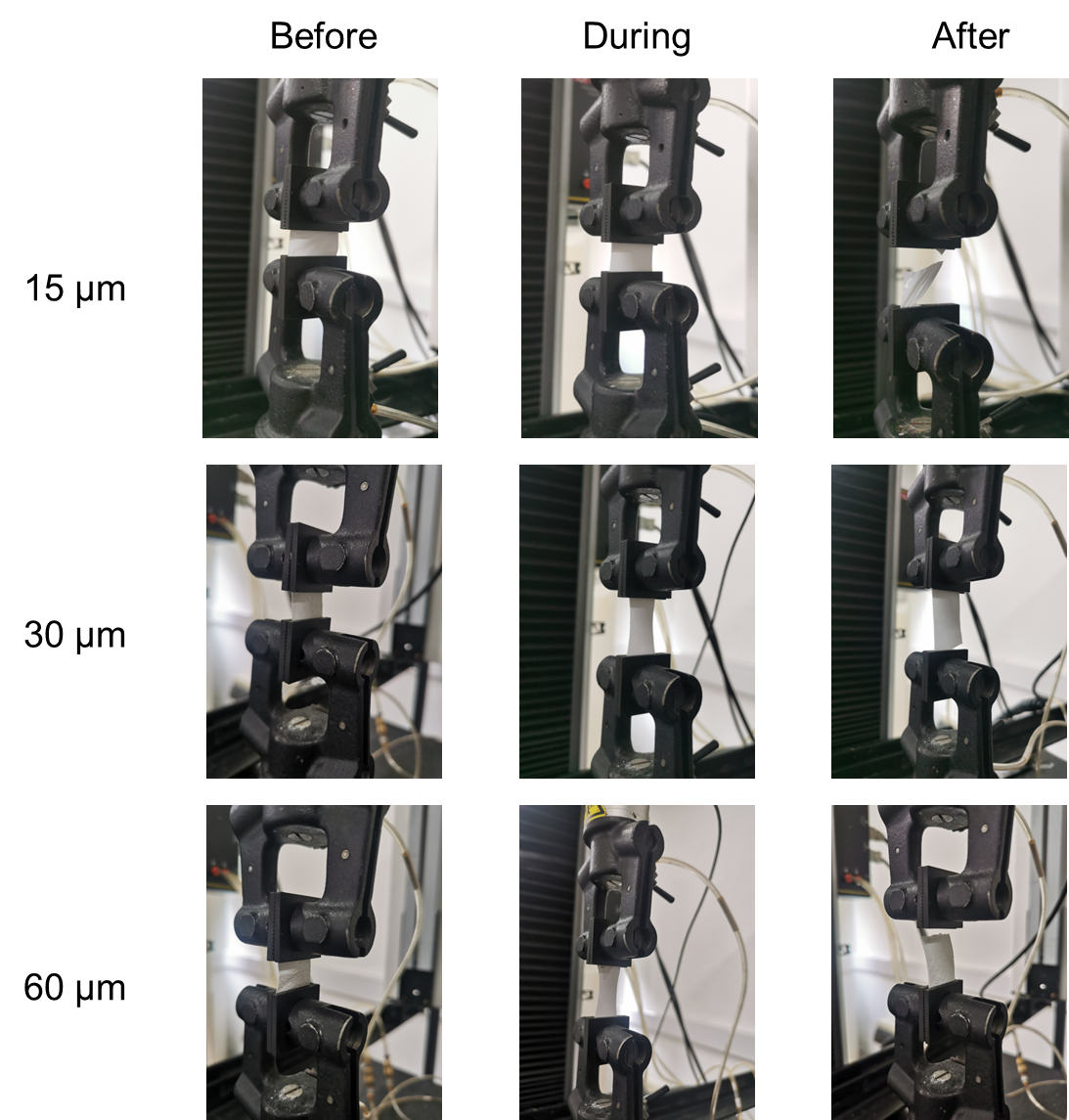
**

Figure S13 The images of tensile test of nanoPE with 15 *μ*m, 30 *μ*m and 60 *μ*m.


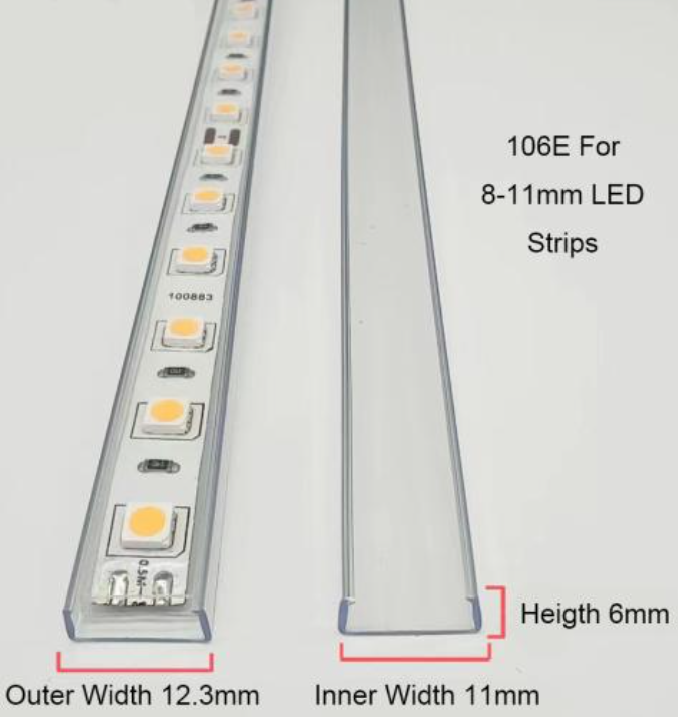


Figure S14 One commercial LED product with PVC cover.

Figure S15 The sustained force of nanoPE with different thicknesses.

**Note S11: Influence of gap between the chip and the cover**

The gap between the chip and the cover influences both the temperature of the LED chip and the illuminated area. In the absence of space between the chip and the cover, a significant portion of the emitted light will be reflected back to the LED chip, as depicted in Fig. S16a, which will heat the LED chip significantly (Fig. S16b).

In our design for streetlight application, we intentionally introduce a gap between the light source and the cover to enable the reflected light to illuminate a larger area on the ground (Fig. S16c). Furthermore, we performed an experiment aiming to verify the correlation between the gap distance and the illuminated area with the standard illuminance higher than 10 lux (i.e. a standard illuminance for parking lots, 1 lux= 0.001496 W/m^2 [R13]^). As shown in Fig. S16d, as the gap distance between the 5cm×5cm LED chip and the cover increases, the illuminated area on the ground increases and saturates at approximately 1.4 m^2^. In our experiment, we selected a gap of 6 cm to balance the temperature, illuminated area, and compactness of the LED package.


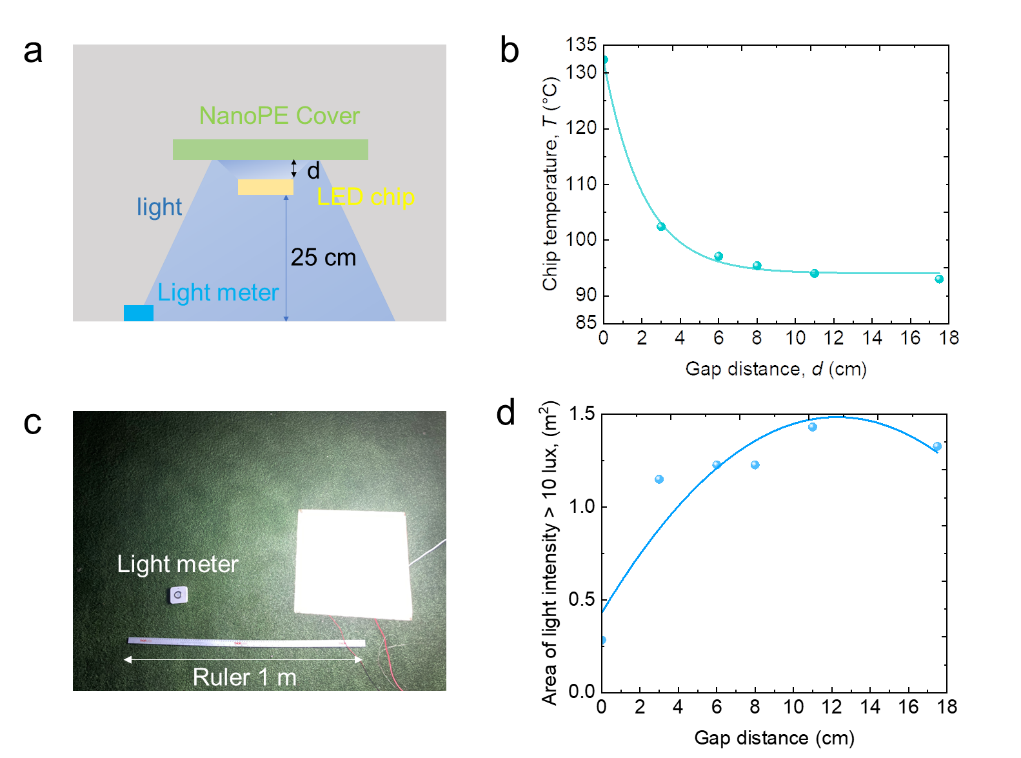


Figure S16 The influence of the gap between the LED chip and the cover. (a) Schematic illustration of the experimental setup. (b) Chip temperature as a function of the gap distance. (c) A photograph of the experimental setup. (d) The illuminated area of illuminance higher than 10 lux as a function of the gap distance.

**Note S12: Detailed experimental settings**

**A: Indoor experimental settings**

To confirm the shift of the wavelength peak caused by the sky-facing radiative cooling, we characterize the spectral property of the output light, using a high-resolution spectrometer (Ocean HR4Pro UV-Vis-ES). To further minimize the influence of ambient radiation, an enclosure box covered with aluminum mylar from the inside was employed. This enclosure box acted as a waveguide, effectively reducing the impact of ambient radiation on the test environment. In this experiment, we employed a direct current (DC) power source to provide a stable and consistent current for the LED chip. The power source was carefully set to a limiting current of 0.2 A to maintain a controlled temperature during the experiment. To accurately measure the temperature of the LED chip and evaluate the radiative cooling performance of our design under different conditions, we positioned a K-type thermocouple at the center of the LED chip. This thermocouple enabled precise and reliable temperature recordings throughout the experiment. In this indoor experiment, nanoPE film with a thickness of 15 *μ*m was used.

To mitigate the cooling effect induced by evaporated liquid nitrogen, we introduced a gap using standard transparent PE and filled it with room-temperature air in our previous setup, as illustrated in Fig. S17. This gap was designed to facilitate thermal radiation heat transfer while effectively isolating the evaporated nitrogen.


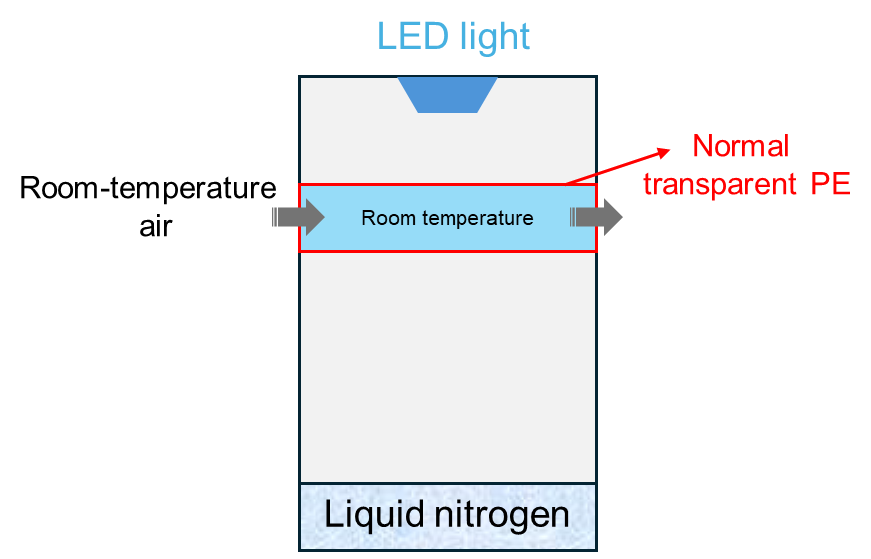


Figure S17 The detailed indoor setup to prevent the cooling effect by evaporated liquid nitrogen.

Commercial LED chips are typically encapsulated with epoxy resin or silicone ^[R12]^, both of which exhibit prominent O-H and C-O bonds, suggesting significant thermal emission in the mid-infrared range ^[R14]^. Here we characterized the thermal emissivity of the front-side of LED chip by a Fourier transform infrared spectrometer (Bruker HYPERION II), with an integrating sphere (A562-G/Q) coated with diffuse gold reflectors, as shown in Fig. S18. As you can see, the average thermal emissivity within 7-13 *μ*m reaches 0.94. Therefore, the proposed sky-facing cooling potential is valid.


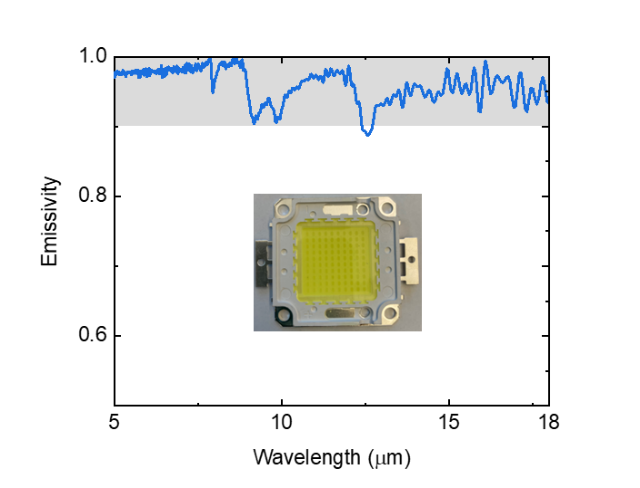


Figure S18 The thermal emissivity of the front-side of LED chip (inset).

**B: Outdoor experimental settings**

In the outdoor experiment shown in Fig. 4d, a nanoPE film with thickness of 15 μm was used and the applied current was also 0.2A. In the outdoor experiment shown in Fig. 5c, nanoPE films with thicknesses from 15 μm to 360 μm were used. In the conducted experiments shown in Fig. 4d and Fig. 5b, double-sided tape was utilized to affix the nanoPE film to the PMMA frame of the customized setup. For the outdoor experiments in Fig. 5d-5f, an optical stand was employed to support this setup.

**Note S13: Characterization of junction temperature**

In addition to measuring the surface temperature of the LED chip (see Note S12), we conducted a specific characterization of the junction temperature, *T*_j_. We utilized an industry-standard photometric testing system (Leets Lighting, LEDT-400A) that incorporates an integrating sphere and a spectrometer (Instrument Systems, CAS 140CT-151), adhering to the IES LM-85-20 standard ^[R15]^ (Fig. S19a).

Within this characterization process, the LED chip was situated within a temperature controller. To maintain a controlled temperature in the system, we administered continuous rated current pulses with a minimal duty cycle of 1% at a defined current. This approach ensured that the LED chip did not experience an excessive temperature rise during testing, allowing the preset temperature in the testing chamber to represent the junction temperature of the LED chip. Notably, under a specified pulsed driving current (for instance, 0.2 A in our experiment), the forward voltage of the LED chip (*V*_f_) demonstrated a direct correlation to *T*_j_. Hence, the relationship observed between *V*_f_ and *T*_j_ (as illustrated in Fig. S19b) facilitated our extraction of the LED chip's junction temperature using the forward voltage. As a result of radiative cooling in the indoor experiment, the junction temperature decreases by 8.6 °C (from 135.0 °C to 126.4 °C).


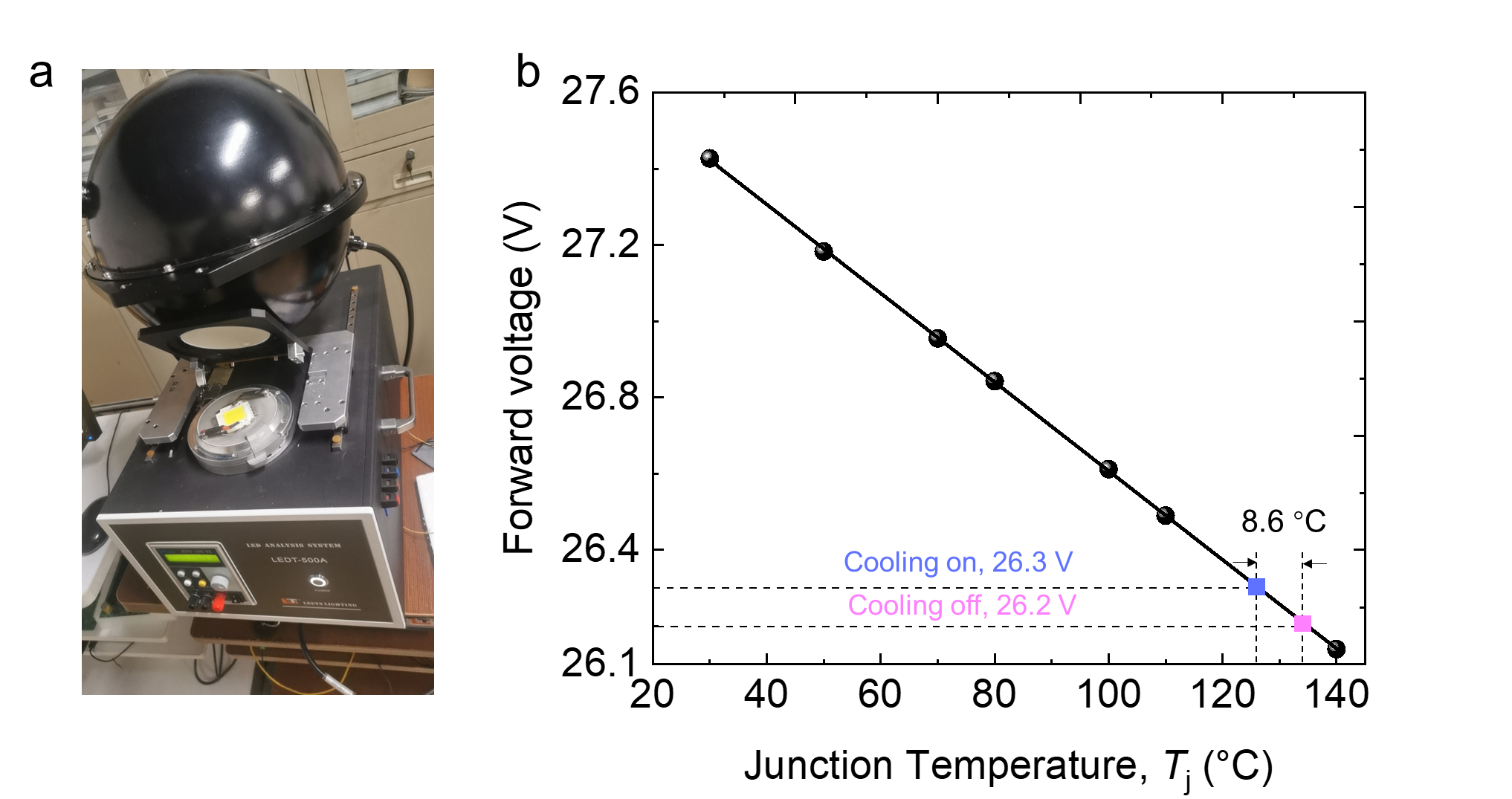


Figure S19 Junction temperature (*T*_j_) characterization and result. (a) The setup for the measurement of optical and electrical properties of the applied LED chip. (b) The forward voltage as a function of *T*_j_ under the current of 0.2 A.

**Note S14: Characterization of emitted light (luminous flux, CCT)**

We used an optic fiber to measure the spectral properties of the emitted light as discussed in Note S12. To capture the emitted light omnidirectionally, we employed a commercialized photometric testing service, utilizing a commercial integrating sphere system (LEDT-400) coupled with another spectrometer (CAS 140CT-151), as depicted in Fig. S19a. The commercial setup enabled us to accurately measure the luminous flux at varying junction temperatures, detailed in Fig. S20a. In this work, we have simulated the sky-cooling effect by analyzing the LED's performance at two distinct temperatures, which correspond to the experiments with or without radiative cooling, as shown in Fig. 3b. This comparison yielded a quantifiable increase in luminous flux of 4.3% (Fig. S20a) with an 8.6 ℃ reduction in junction temperature, as illustrated in Fig. S20b.


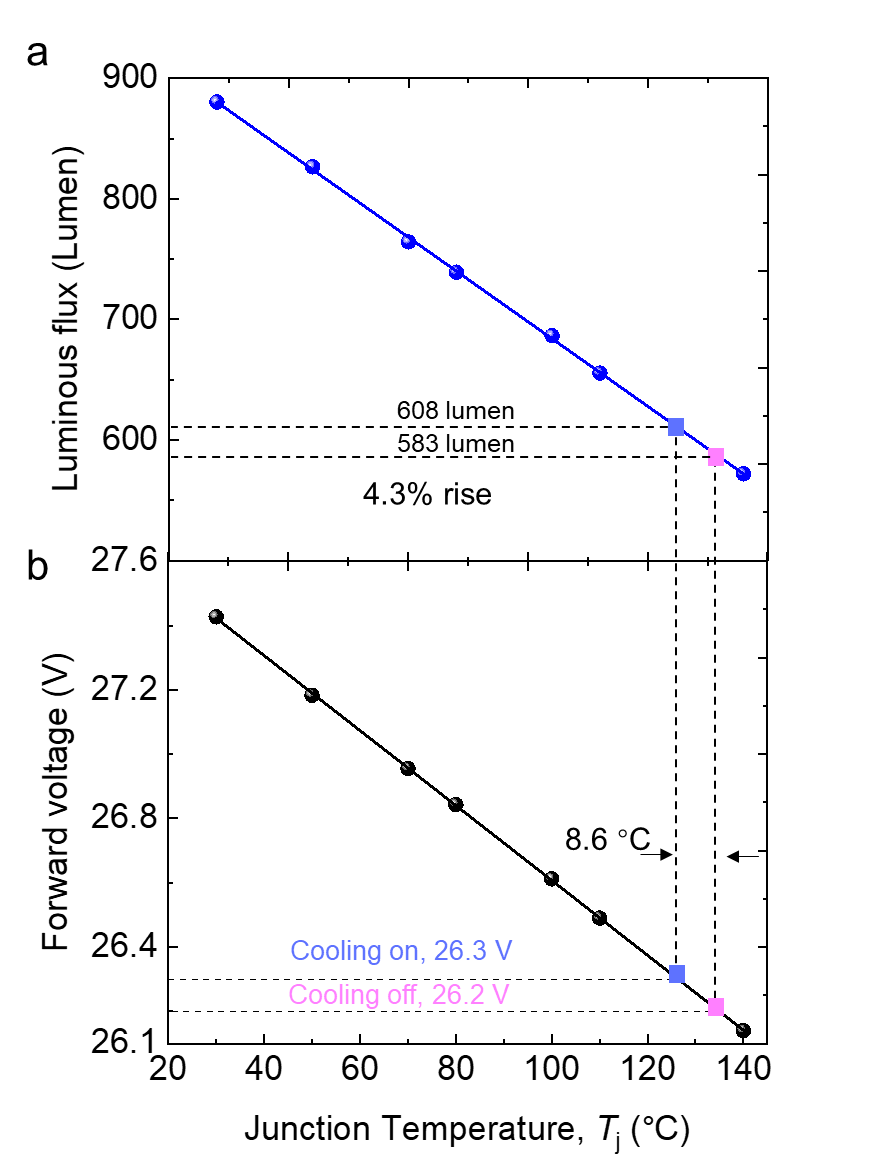


Figure S20 (a) The luminous properties of the applied LED chip under 0.2 A and different junction temperatures. (b) The forward voltage as a function of junction temperature. The indoor experiment resulted in an 8.6 ℃ reduction in junction temperature due to radiative cooling.

**Correlated Color Temperature (CCT) ^[R16]^**: According to the spectral properties of the emitted light, which is measured by the spectrometer, one can determine the Correlated Color Temperature (CCT). The CCT is a measure of the color appearance of light, expressed in Kelvin. It characterizes the color of light emitted by a light source, indicating whether the light appears warm, neutral, or cool. Lower CCT values (e.g., 2700 K) correspond to warm or yellowish light, while higher CCT values (e.g., 5000K and above) indicate cooler or bluish light. The term "correlated" implies that the color temperature is an approximate value, as the color of light can vary and is not strictly correlated with the temperature of an ideal black-body radiator that emits light of similar color. The calculation is as follows. Firstly, we can compute the tristimulus values (*X*, *Y*, *Z*) by

$X=\int I\left(\lambda\right)\overline{x}\left(\lambda\right)d\lambda$ (S14)

$Y=\int I\left(\lambda\right)\overline{y}\left(\lambda\right)d\lambda$ (S15)

$Z=\int I\left(\lambda\right)\overline{z}\left(\lambda\right)d\lambda$ (S16)

Parameters $\overline{x}$, $\overline{y}$ and $\overline{z}$ represent the CIE [color matching functions](https://www.sciencedirect.com/topics/engineering/color-matching-function), respectively ^[R23]^. After calculating *X*, *Y*, and *Z*, the coordinates CIEx and CIEy are obtained by

$\text{CIEx=}\frac{X}{X+Y+Z}$ (S17)

$\text{CIEy=}\frac{Y}{X+Y+Z}$ (S18)

Based on the calculated CIE xy coordinate values, the CCT value can be obtained by plotting these coordinates on a standard CIE chart ^[R18, R19]^.

**Note S15:** **The results of spectrometer**

Upon referencing the product manual of the portable high-resolution fiber-based spectrometer (HR4Pro UV-Vis-ES) utilized in our study, we discovered that this commercial instrument's thermal stability registers an error of 0.3 pixel/ºC ^[R20]^. Initially, our experiments were conducted at different ambient temperatures: i.e., indoors at 21 ºC and outdoors at 27.5 ºC. Consequently, the 6.5 ºC temperature disparity between the indoor and outdoor settings could lead to a spectral instability of around 0.4 nm, accounting for the spectrometer's accuracy of approximately 0.2 nm/pixel (as specified in the calibration data sheet). To avoid this instability, we conducted an additional outdoor experiment (Fig. S21a) under a controlled ambient temperature of 21 ºC, closely resembling the indoor conditions. The results, illustrated in Fig. S21b, elucidate a temperature decrease of 3.9 ºC due to radiative cooling, resulting in a blue peak shift of 0.2 nm (as observed in Fig. S21c and S21d). This outcome aligns with the findings from the indoor experiment (7.6 ℃, 0.4 nm).


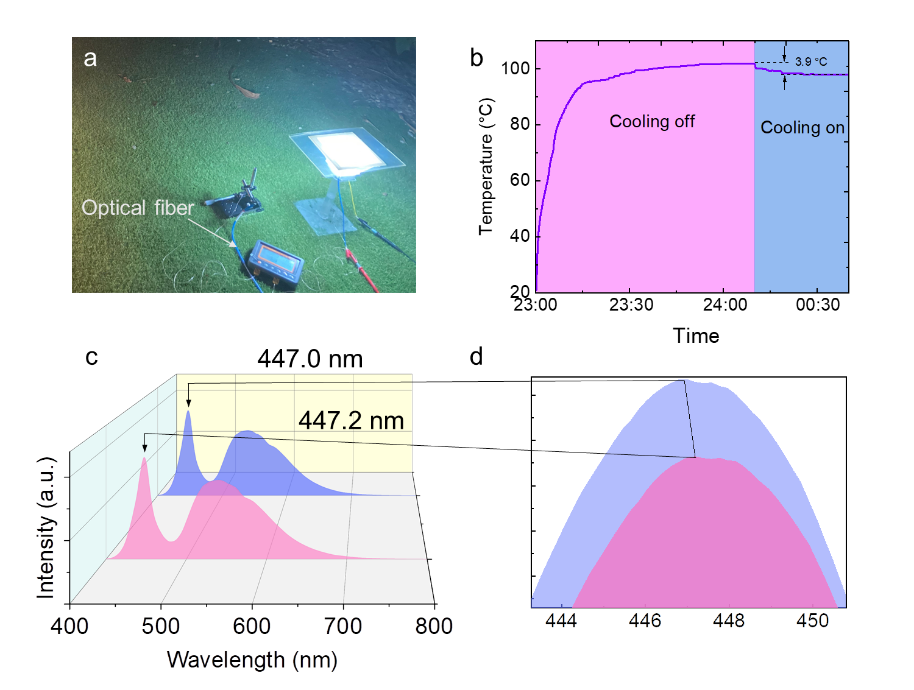


Figure S21 (a) The outdoor experiment demonstration under an ambient temperature of ~21 ℃. (b) The temperature of the LED chip under cooling off and cooling on. (c) The spectral properties of the emitted light under conditions of “cooling on” and “cooling off”. (d) Zoom-in view of the blue peak.

**Note S16: Examination of the spectral characteristics determined through optical fiber measurements**

To measure the relative increased efficiency in the LED performance when its temperature is lowered, the measured intensity curves were multiplied by the photopic luminous function^[R21]^ to adjust the intensity based on human eye sensitivity to different visible wavelengths. Then, the area under each adjusted intensity curve was divided by the input power and the ratio of the resulting values for each cooling mode is used to determine the increase in efficiency of the LED in “cooling on” mode relative to the “cooling off” mode. Although this calculation does not provide the absolute optical power of each mode, it still provides a correct relative measurement as measurements taken with a single spectrometer are accurate relative to one another^[R22]^. A similar calculation can be found in Ref. [R23] and Ref. [R24]. The process of the LED efficiency calculation is summarized in the below equation:

$\eta=\frac{\int_{380}^{780} f_{\text{On}}\left(\lambda\right)V\left(\lambda\right)d\lambda}{\int_{380}^{780} f_{\text{Off}}\left(\lambda\right)V\left(\lambda\right)d\lambda}\times\frac{P_{\text{In-Off}}}{P_{\text{In-On}}}$ (S19)

Where

• *f*_On_(λ)is the spectral intensity in “cooling on” mode.

• *f*_Off_(λ)is the spectral intensity in “cooling off” mode.

• *V*(λ) is the photopic luminous function, which can be found in Ref. [R17].

• *P*_In-On_ is the input power in “cooling on” mode.

• *P*_In-Off_ is the input power in “cooling off” mode.

To perform these calculations, Matlab was used and the calculations showed a relative efficiency increase of ~4.9% in the indoor experiment and ~4.4% in the outdoor experiment (clean sky).

**Note S17: Weather data on the days of outdoor experiment under clean sky and under cloudy sky**


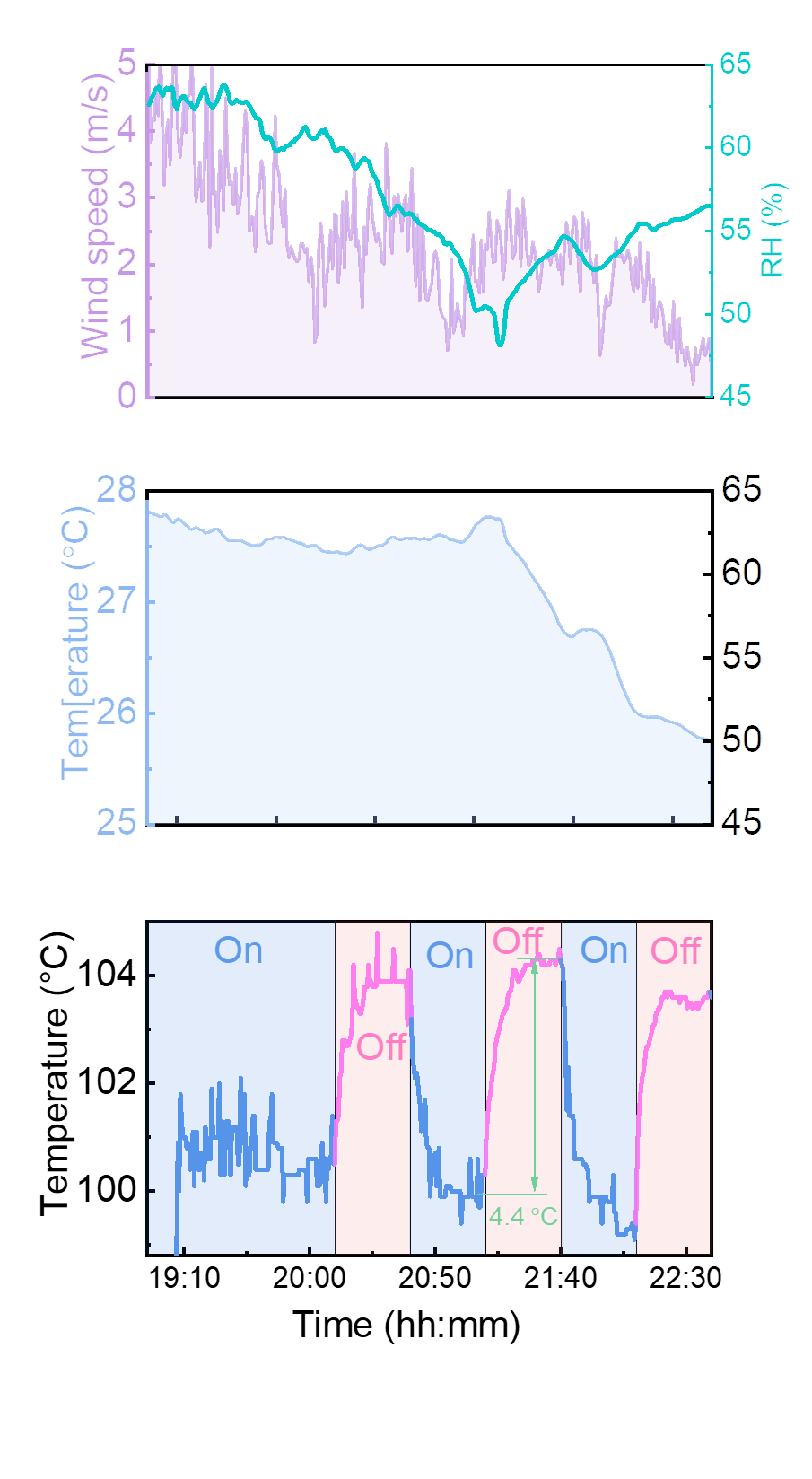


Figure S22 The outdoor temperature and wind speed during the operating of outdoor experimental test under clean sky.


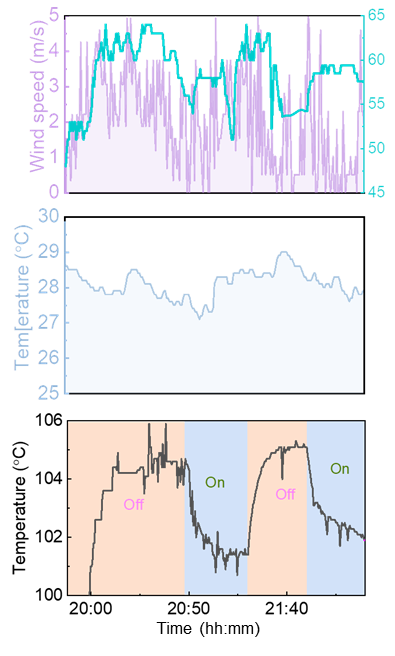


Figure S The outdoor temperature and wind speed during the operating of outdoor experimental test under cloudy sky.

**Note S18: Radiative cooling effect on a commercial LED lamp**

We selected a commercial outdoor LED product with a sky-facing LED installed inside the light housing ^[R25]^ (see top left panel in Fig. S24a) to further characterize the bulb's power, color temperature, and illuminated area by using the proposed nanoPE ceiling. This LED fixture incorporates a sky-facing LED chip (from the LEDT-12WWG2 bulb ^[R26]^) with a color temperature of ~3000 K. To quantitatively compare its performance, we assessed parameters including correlated color temperature (CCT), electricity usage, chip temperature, light intensity, and the illuminated area using different covers. In this work, we've incorporated three covers: the original black cover (top right panel), the nanoPE+PMMA cover (cooling off, bottom left panel), and the nanoPE cover (cooling on, bottom right panel) under identical driving conditions (i.e., 220 V 50Hz). However, due to the original black cover's strong light absorption, the ground light intensity from the lamp with this cover is considerably lower compared to the lamp with the reflective nanoPE cover (refer to Fig. S24b). Hence, comparing the lamp with the original cover to the other two covers would be unfair. Thus, our focus is on examining the performance of the lamp with cooling-on and cooling-off covers to assess the impact of radiative cooling. As shown in Fig. S24c, when changing from the cooling-off cover to cooling-on cover under the same input power of 11.8 W, the lamp's temperature decreased by 3.7 ℃ due to the activated radiative cooling. Due to this temperature reduction, the CCT will reduce from 3034 K to 3025 K (see technical details in Note S14). By measuring the spectral light intensity using a fiber-based spectrometer (Fig. S24b), the cooling-on cover also resulted in an approximately 7% increase in light intensity compared to the cooling-off cover.

To evaluate the illuminated area with a standard light intensity higher than 10 lux (typical for parking lots), we conducted an extra experiment as shown in Fig. S24d. We utilized a light meter (FUTUREHORTI) positioned on the ground to measure the radius at locations with an illuminance of 10 lux. Subsequently, we computed the area corresponding to this radius. Due to radiative cooling, the illuminated area with "cooling on" (5.0 m^2^) was 6% larger than with "cooling off" (5.3 m^2^).


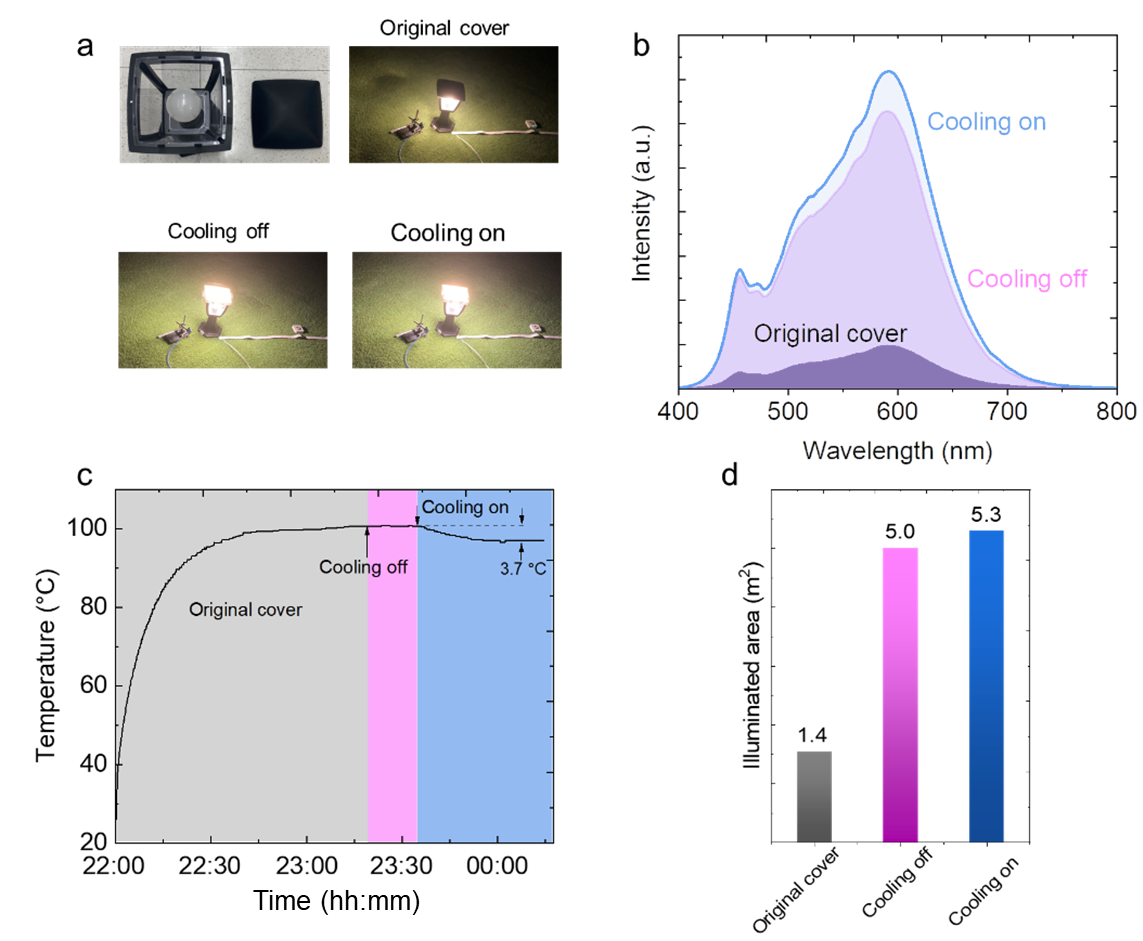


Figure S24 (a) The experiment demonstration of the commercial LED product with original cover, cooling-off cover and cooling-on cover. (b) The spectral properties of the emitted light with three covers. (c) The chip surface temperature of this LED product. (d) The illuminated area of illuminance higher than 10 lux.

**Note S19: Characterization of the LED light under cloudy sky**

The measured spectral intensity of the LED light under cloudy sky is shown in Fig. S25. The blue peak of the LED chip shifted from 446.6 nm to 446.4 nm under the cloudy sky, confirming the reduced operational temperature of the LED chip. According to Note S14, the luminous flux is increased by 3.1% after using the sky for cooling. The junction voltage increases from 26.07 V to 26.15 V (Fig. S26). Therefore, the efficiency of the LED light under cloudy sky with sky cooling is increased by 3.1%.


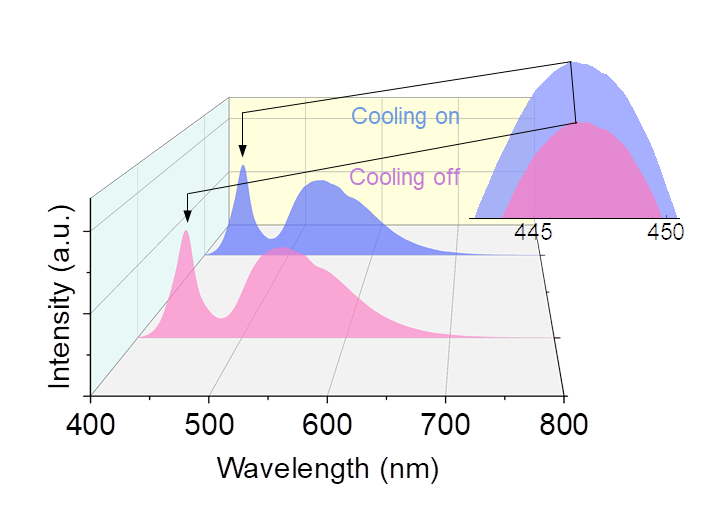


Figure S25 The spectral intensity of the emitted light from LED chip under clean sky.


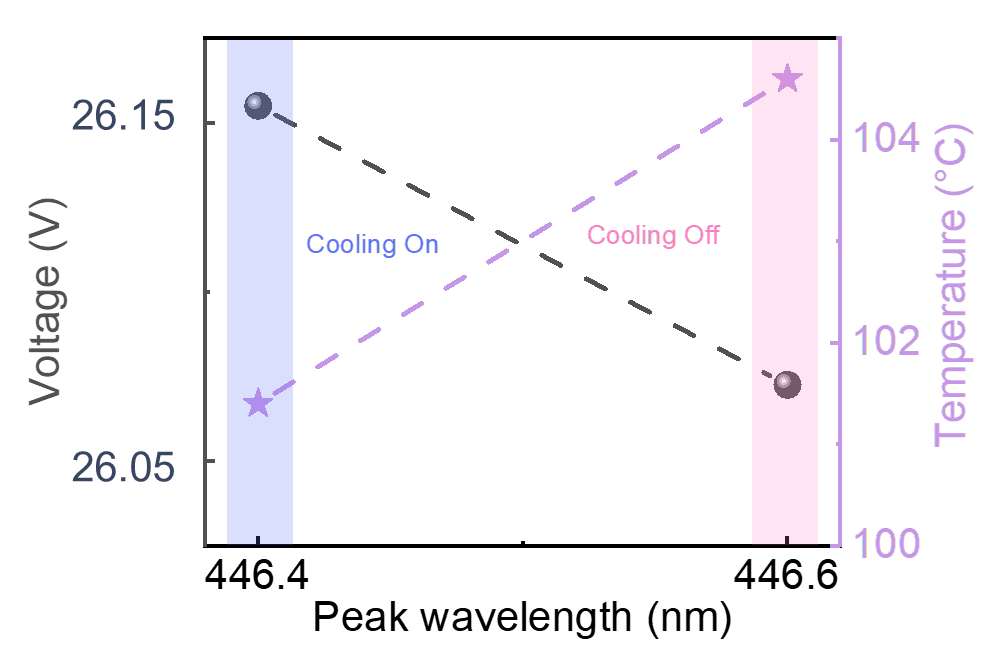


Figure S26 Voltage and temperature vs peak wavelength at cooling on and cooling off under cloudy sky.

**Note S20: Cooling power calculation for the indoor and outdoor experiments**

According to the calculations in Note S2, the net cooling powers of LED chip facing sky with cooling channel under indoor and outdoor experiment conditions can be obtained based on the corresponding temperatures and the optical properties of the nanoPE cover. The averaged thermal reflectance, transmittance and emittance of the applied nanoPE in the experiments are 0.05, 0.05 and 0.9. The temperature of the LED chip and the surface temperature of the cover at indoor experiment are 92.3 °C and 31.1 °C. The corresponding cooling power by radiative heat transfer is 835.4 W/m^2^ according to Eq. (S11). The ambient is 20 °C. Based on a non-radiative heat transfer coefficient of *h*_com_=8.3 W/(m^2^∙K), which is obtained by

$h_{\text{c-g}}=8.3+2.5V_{w},V_{w}<8\text{m}/\text{s}$ (S19)^[R4]^

the non-radiative cooling power for the indoor experiment is 600.1 W/m^2^. Therefore, the net cooling power is 1435.5 W/m^2^. For outdoor experiment under clean sky, the temperatures of ambient and the nanoPE cover and the chip are 27.5 °C, 34.1 °C and 99.8 °C, respectively. The corresponding cooling power by radiation is 727.2 W/m^2^ according to Eq. (S11). With an averaged wind speed *V*_w_= 2.8 m/s, the non-radiative heat transfer coefficient is *h*_c-g_=15.3 W/(m^2^∙K) and the non-radiative cooling power is 1106.2 W/m^2^. The calculated net cooling power is 1833.4 W/m^2^. For outdoor experiment under cloudy sky, the temperatures of ambient and the nanoPE cover and the chip are 28.1 °C, 34.3 °C and 101.4 °C, respectively. The corresponding cooling power by radiation is 698.7 W/m^2^ according to Eq. (S11). With an averaged wind speed *V*_w_= 2.7 m/s, the non-radiative heat transfer coefficient is *h*_c-g_=15.0 W/(m^2^∙K) and the non-radiative cooling power is 1099.5 W/m^2^. The calculated net cooling power is 1798.2 W/m^2^.

**Note S21: Visible transmission of nanoPE**

The spectral visible transmission of nanoPE films with different thicknesses can be found in Fig. S27. According to Eq. (S13), one can obtain the average visible transmission.

Figure S27 The spectral visible transmission.

**Note S22: Relationship between luminosity and chip surface temperature**

The benefit of the nanoPE film was meticulously evaluated by charting the luminous flux (lumens) as a function of the surface temperature of the LED chip, which operates at a current of 0.1A. Utilizing a commercial temperature-controllable integrating sphere system, this function was characterized as depicted in Fig. S28. Through precise temperature adjustments, we established a quantitative relationship demonstrating the increase in luminosity corresponding to a decrease in surface temperature.

**
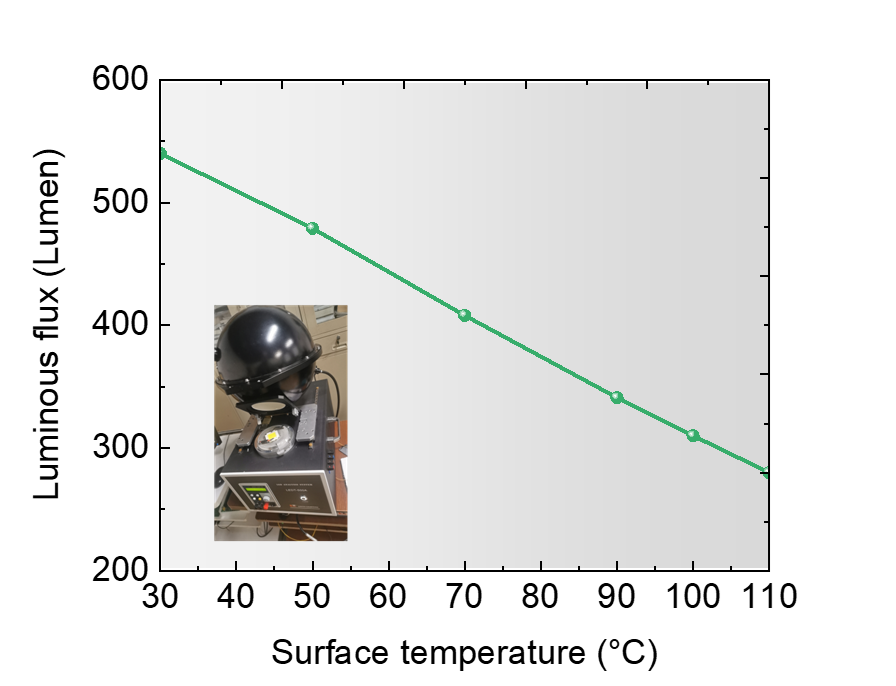
**

Figure S28 The characterized luminous flux (lumens) using an integrating sphere (inset) as the function of the surface temperature of the LED chip.

**Note S23: Effect on the distribution of light intensity**

As shown in **Figure S29**, the illuminance of a ground-facing LED chip decreases rapidly from the center of the illuminated area, whereas the sky-facing LED with nanoPE provides a more consistent light distribution. As a result, the area illuminated with an illuminance higher than 10 lux (typically required for parking lots ^[R13]^) increased by 74% due to the reflective nanoPE cover. Additionally, the illuminated area expanded by a further 4% when radiative cooling was active compared to when it was not.


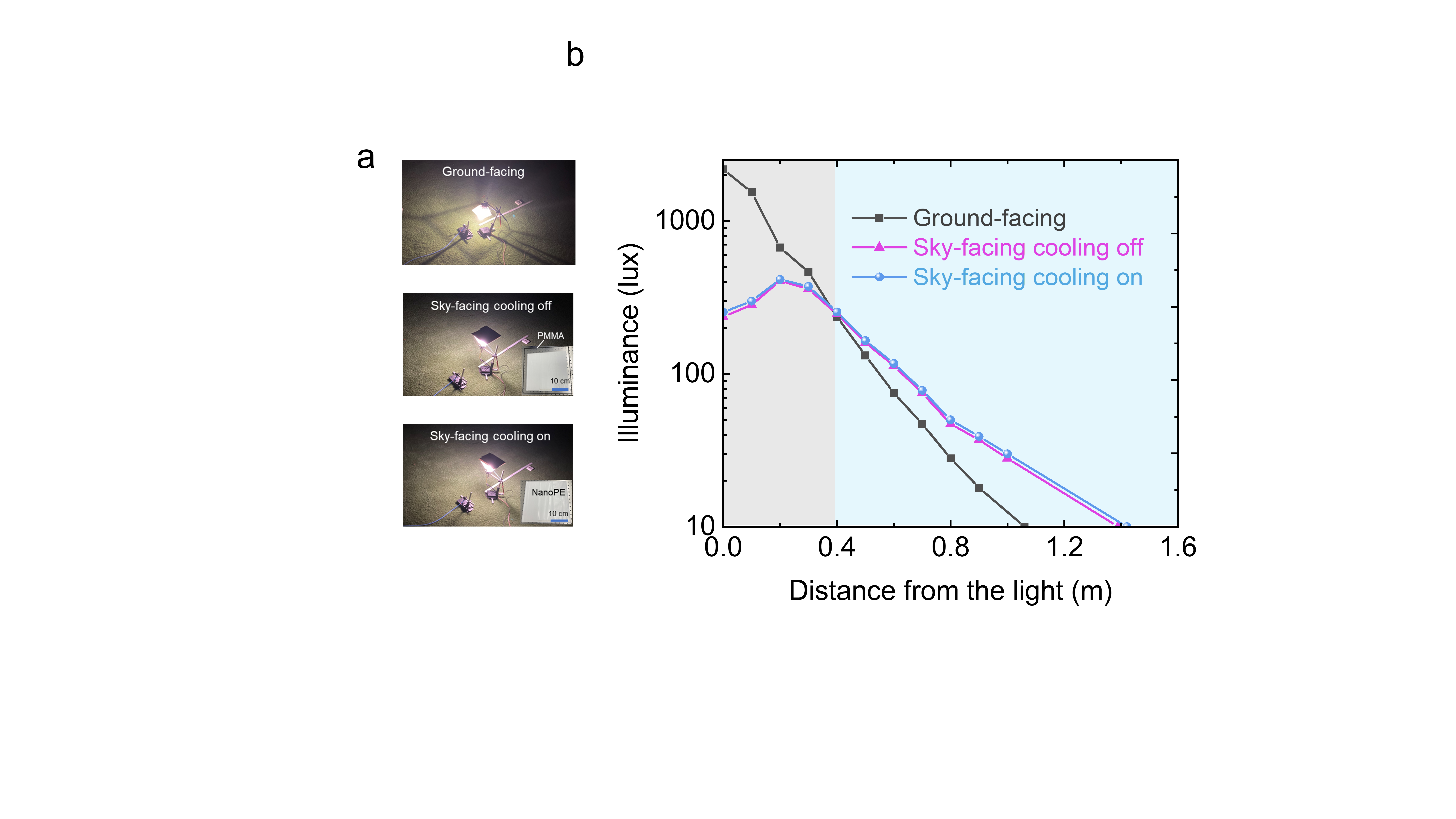


Figure S29 Illuminance as a function of horizontal distance from the LED chip.

**Note S24: More details about the experiment under extreme environments and practical conditions**

The main objective of the experiment was to compare the temperatures achieved in two scenarios: (1) When the LED was intercepted by a layer of 360-µm-thick nanoPE film (cooling on), the system would allow thermal transmission. (2) When the LED was intercepted by both nanoPE and PMMA films (cooling off), the thermal radiation would be blocked while not affecting visible light illumination. By analyzing the temperature differences under these two different conditions, we were able to assess the effectiveness of our design in enhancing heat dissipation. These outdoor experiments (Figure S30) were conducted on the KAUST campus on the evenings of July 21 and 22, 2024, respectively. In this experiment, the extreme environments are realized by a chiller which can control the temperature between 0-50 ℃. A temperature probe is put at the center of the insulation box to confirm its temperature as an environment temperature.

**
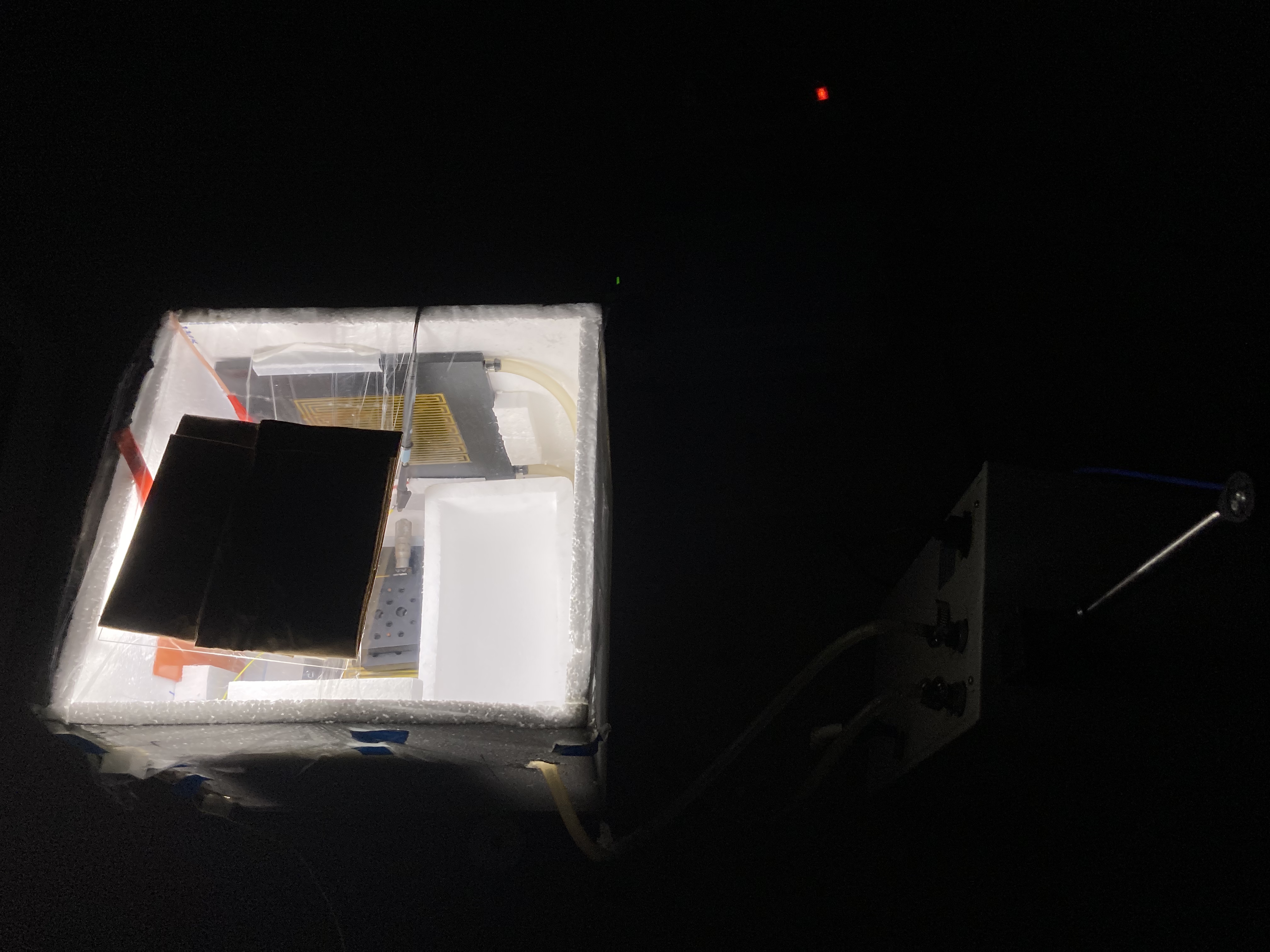
**

Water circulation pipe

Chiller

Figure S30 Outdoor experiment under extreme environments.

**Self-cleaning effect from hydrophobic surface**

The contact angle was measured using a DSA100E with a 2 μL droplet, as shown in Figure S31. Due to the large contact angle, water easily slips off the surface of the nanoPE film, effectively carrying away any dust and keeping the film clean.

**
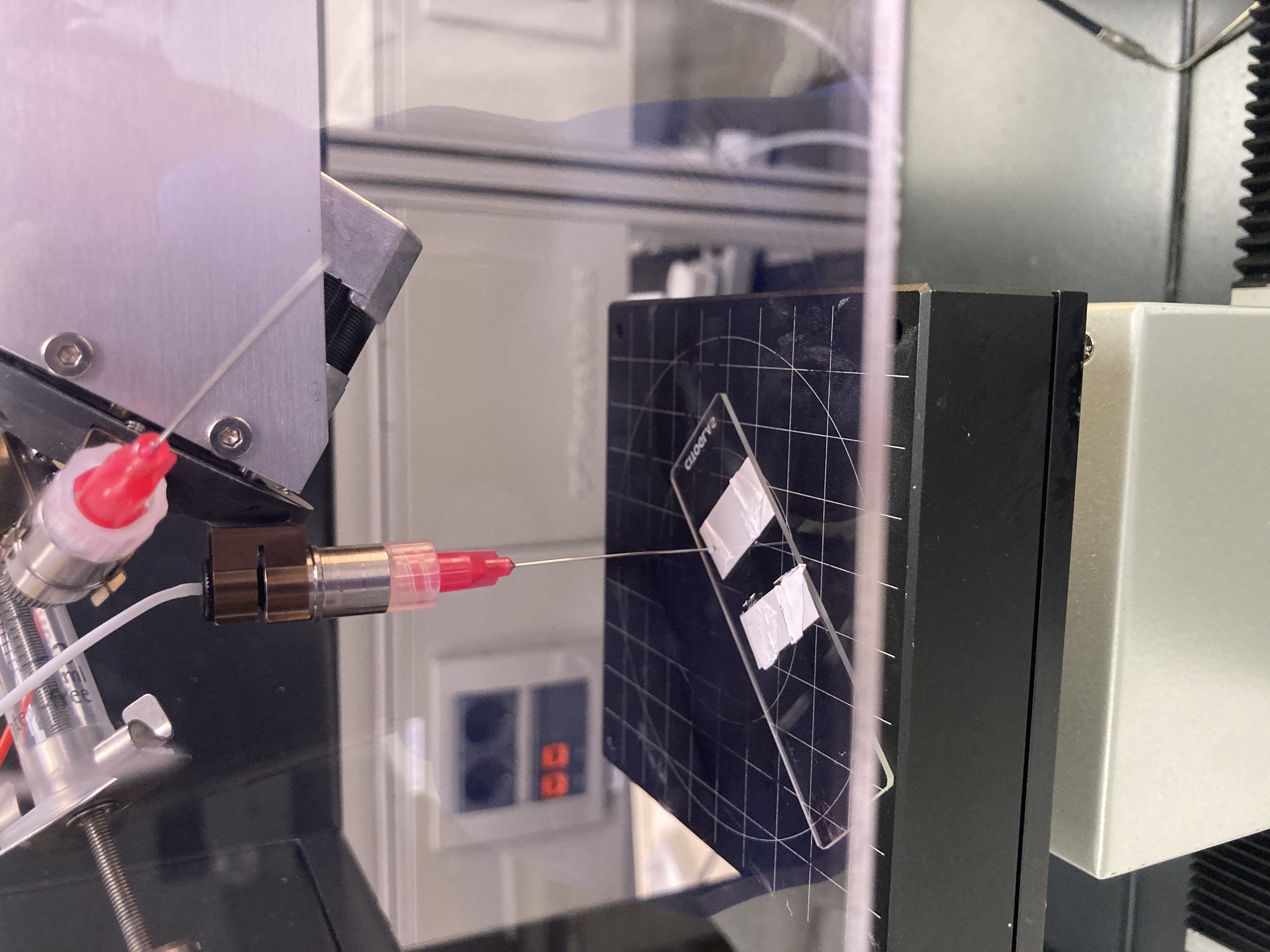
**

Figure S31 The characterization of contact angle.

**Note S25: More discussion about the energy saving estimation**

In this work, we estimated the potential benefits of applying radiative cooling technology to outdoor LED lighting systems utilizing data on the total annual energy consumption attributed to outdoor LED lighting across the US—specifically, 76.9 terawatt-hours (TWh) ^[R27]^: i.e., should the suggested sky-facing radiative cooling design be universally implemented in place of the current outdoor LED lighting fixtures in the U.S., the anticipated energy savings could reach an impressive 1.9 TWh (calculated as 76.9 TWh ×2.5%).

**Reference**

[R1] Bergman, T. L., Incropera, F. P., DeWitt, D. P., & Lavine, A. S. (2011). Fundamentals of heat and mass transfer. John Wiley & Sons.

[R2] Raman, A. P., Anoma, M. A., Zhu, L., Rephaeli, E., & Fan, S. (2014). Passive radiative cooling below ambient air temperature under direct sunlight. Nature, 515(7528), 540-544.

[R3] Lord, S.D., 1992. A New Software Tool for Computing Earth's Atmospheric Transmission of Near- and Far-Infrared Radiation. NASA Technical Memorandom 103957. Data available from Gemini Observatory: IR Transmission Spectra, <https://www.gemini.edu/sciops/telescopes-and-sites/observing-condition-constraints/ir-transmission-spectra

[R4] Zhao, D., Aili, A., Zhai, Y., Lu, J., Kidd, D., Tan, G., Yin, X., & Yang, R. (2019). Sub-ambient cooling of water: Toward real-world applications of daytime radiative cooling. Joule, 3(1), 111-123.

[R5] Bohren, C. F., & Huffman, D. R. (2008). Absorption and scattering of light by small particles. John Wiley & Sons.

[R6] Refractive index of polymer. Retrieved from: <https://scipoly.com/technical-library/refractive-index-of-polymers-by-index/>

[R7] M. David, D. Disnan, A. Lardschneider, D. Wacht, H. Hoang, G. Ramer, H. Detz, B. Lendl, U. Schmid, G. Strasser, B. Hinkov. Structure and mid-infrared optical properties of spin-coated polyethylene films developed for integrated photonics applications, Opt. Mater. Express 12, 2168-2180 (2022)

[R8] Ambroz, F., Macdonald, T. J., Martis, V., & Parkin, I. P. (2018). Evaluation of the BET Theory for the Characterization of Meso and Microporous MOFs. Small methods, 2(11), 1800173.

[R9] Su, K., Tao, Y., & Zhang, J. (2021). Highly transparent plasticized PVC composite film with ideal ultraviolet/high-energy short-wavelength blue light shielding. Journal of Materials Science, 56(30), 17353-17367.

[R10] Yin, X., Zhang, Y., & Zhang, J. (2017). Transparent plasticised PVC film with ultraviolet and high-energy visible light shielding performance. Plastics, Rubber and Composites, 46(9), 375-380.

[R11] Clear Plastic LED Channel for Strip Lights. Retrieved from <https://www.ledbe.com/clear-plastic-led-channel-for-strip-lights?srsltid=AfmBOoojlMmRvE3h2U47oipyHc_fsya-AS74BJh4ZGw1L9wT9M2Msg8yLKU>

[R12] Lu, D., & Wong, C. P. (Eds.). (2009). Materials for advanced packaging (Vol. 181). New York: Springer.

[R13] Rich, C., & Longcore, T. (Eds.). (2013). Ecological consequences of artificial night lighting. Island Press.

[R14] Zhou, Z., Wang, X., Ma, Y., Hu, B., & Zhou, J. (2020). Transparent polymer coatings for energy-efficient daytime window cooling. Cell Reports Physical Science, 1(11).

[R15] IES LM-85-20. Approved Method: Optical and Electrical Measurements of LED Packages and Arrays. STANDARD by Illuminating Engineering Society , 02/07/2020

[R16] CIE Colorimetry - Part 2: Standard Illuminants for Colorimetry, 2nd Edition

[R17] Yalçın, R. A., Blandre, E., Joulain, K., & Drévillon, J. (2020). Colored radiative cooling coatings with nanoparticles. ACS photonics, 7(5), 1312-1322.

[R18] Hasabeldaim, E. H. H., Swart, H. C., & Kroon, R. E. (2023). Luminescence and stability of Tb doped CaF2 nanoparticles. RSC advances, 13(8), 5353-5366.

[R19] Calculate color temperature (CCT) from CIE 1931 xy coordinates. Retrieved from:

https://www.waveformlighting.com/tech/calculate-color-temperature-cct-from-cie-1931-xy-coordinates

[R20] HR4Pro High-resolution Spectrometers. Retrieved from: https://www.oceaninsight.com/globalassets/catalog-blocks-and-images/pdfs/hr4pro_product-sheet.pdf

[R21] CIE 1988 2° Spectral Luminous Efficiency Function for Photopic Vision. CIE; 1990. 11 p.

[R22] FAQ on spectroscopy measurements | Ocean Insight. Retrieved from: https://www.oceaninsight.com/support/faqs/measurements/

[R23] Luminous Efficacy Radiation in LED System Calculations. 2016. Retrieved from: <https://www.youtube.com/watch?v=Lqbyu5BGthc>

[R24] IEC 60050 - International Electrotechnical Vocabulary - Details for IEV number 845-21-045: “luminous intensity”. Retrieved from: <https://www.electropedia.org/iev/iev.nsf/display?openform&ievref=845-21-045>

[R25] Commercial outdoor LED product. Retrieved from: [https://item.taobao.com/item.htm?spm=a21n57.1.0.0.4213523cQHbnMZ&id=546186689418&ns=1&abbucket=16#](https://item.taobao.com/item.htm?spm=a21n57.1.0.0.4213523cQHbnMZ&id=546186689418&ns=1&abbucket=16)

[R26] Commercial LED bulb. Retrieved from: <https://www.amazon.sa/-/en/Alfanar-12W-Warm-White-Bulb/dp/B0BS1GVDMW/ref=sr_1_9?keywords=led+bulb&qid=1704111349&sr=8-9>

[R27] U.S. Department of Energy (2020), Adoption of Light-Emitting Diodes in Common Lighting Applications. Retrieved from <https://www.energy.gov/eere/ssl/led-adoption-report>.
